# Plasma p‐tau181 and GFAP reflect 7T MR‐derived changes in Alzheimer's disease: A longitudinal study of structural and functional MRI and MRS

**DOI:** 10.1002/alz.14318

**Published:** 2024-11-19

**Authors:** Laura Göschel, Andrea Dell'Orco, Ariane Fillmer, Semiha Aydin, Bernd Ittermann, Layla Riemann, Sylvain Lehmann, Stefan Cano, Jeanette Melin, Leslie Pendrill, Patty L. Hoede, Charlotte E. Teunissen, Claudia Schwarz, Ulrike Grittner, Péter Körtvélyessy, Agnes Flöel

**Affiliations:** ^1^ Department of Neurology Charité ‐ Universitätsmedizin Berlin, Corporate Member of Freie Universität Berlin and Humboldt‐Universität zu Berlin Berlin Germany; ^2^ Neuroscience Clinical Research Center Charité ‐ Universitätsmedizin Berlin, Corporate Member of Freie Universität Berlin and Humboldt‐Universität zu Berlin Berlin Germany; ^3^ Physikalisch‐Technische Bundesanstalt (PTB), Braunschweig and Berlin Berlin Germany; ^4^ Department of Neuroradiology Charité ‐ Universitätsmedizin Berlin, Corporate Member of Freie Universität Berlin and Humboldt‐Universität zu Berlin Berlin Germany; ^5^ Institute for Applied Medical Informatics Center for Experimental Medicine University Medical Center Hamburg‐Eppendorf (UKE) Hamburg Germany; ^6^ LBPC‐PPC Université de Montpellier INM INSERM, IRMB CHU de Montpellier Montpellier France; ^7^ Modus Outcomes Ltd Cheltenham UK; ^8^ Division Safety and Transport Division Measurement Science and Technology RISE, Research Institutes of Sweden Gothenburg Sweden; ^9^ Neurochemistry Laboratory Department of Laboratory Medicine Amsterdam Neuroscience Amsterdam UMC Vrije Universiteit Amsterdam Amsterdam the Netherlands; ^10^ Department of Neurology University Medicine Greifswald Greifswald Germany; ^11^ Institute of Biometry and Clinical Epidemiology Charité ‐ Universitätsmedizin Berlin, Corporate Member of Freie Universität Berlin and Humboldt‐Universität zu Berlin Berlin Germany; ^12^ German Center for Neurodegenerative Diseases (DZNE) Standort Magdeburg Magdeburg Germany; ^13^ German Center for Neurodegenerative Diseases (DZNE) Standort Rostock/Greifswald Greifswald Germany

**Keywords:** 7 Tesla, Alzheimer's disease, amyloid beta 42/40, blood‐based biomarkers, functional magnetic resonance imaging, glial fibrillary acidic protein, memory, mild cognitive impairment, magnetic resonance imaging, magnetic resonance spectroscopy, neurofilament light chain, NeuroMET Memory Metric, plasma biomarkers, subjective cognitive decline, tau phosphorylated at threonine 181

## Abstract

**BACKGROUND:**

Associations between longitudinal changes of plasma biomarkers and cerebral magnetic resonance (MR)‐derived measurements in Alzheimer's disease (AD) remain unclear.

**METHODS:**

In a study population (*n* = 127) of healthy older adults and patients within the AD continuum, we examined associations between longitudinal plasma amyloid beta 42/40 ratio, tau phosphorylated at threonine 181 (p‐tau181), glial fibrillary acidic protein (GFAP), neurofilament light chain (NfL), and 7T structural and functional MR imaging and spectroscopy using linear mixed models.

**RESULTS:**

Increases in both p‐tau181 and GFAP showed the strongest associations to 7T MR‐derived measurements, particularly with decreasing parietal cortical thickness, decreasing connectivity of the salience network, and increasing neuroinflammation as determined by MR spectroscopy (MRS) myo‐inositol.

**DISCUSSION:**

Both plasma p‐tau181 and GFAP appear to reflect disease progression, as indicated by 7T MR‐derived brain changes which are not limited to areas known to be affected by tau pathology and neuroinflammation measured by MRS myo‐inositol, respectively.

**Highlights:**

- This study leverages high‐resolution 7T magnetic resonance (MR) imaging and MR spectroscopy (MRS) for Alzheimer's disease (AD) plasma biomarker insights.
- Tau phosphorylated at threonine 181 (p‐tau181) and glial fibrillary acidic protein (GFAP) showed the largest changes over time, particularly in the AD group.
- p‐tau181 and GFAP are robust in reflecting 7T MR‐based changes in AD.
- The strongest associations were for frontal/parietal MR changes and MRS neuroinflammation.

## 1 INTRODUCTION

Due to their limited accessibility, high cost, and risk profile, alternatives to biomarkers measured in cerebrospinal fluid (CSF) or by positron emission tomography (PET) are needed for earlier diagnosis as well as monitoring the effects of disease‐modifying treatments in Alzheimer's disease (AD). Plasma biomarkers are suitable for identifying amyloid or tau pathology, and cognitive decline.^1^, ^2^, ^3^ Therefore, the Alzheimer's Association encourages the careful usage of plasma biomarkers for categorization, diagnosis, and staging.^4^, ^5^

There is evidence indicating that the plasma amyloid beta (Aß)42/40 ratio decreases in incipient stages of the disease and quickly reaches a plateau.^3^ The cause of this plateau remains unclear and there is concern that the initial decrease might be too subtle on an individual level and hidden behind low accuracy for specific measurement methods of Aß42/40.^6^ Complementary information is therefore necessary to draw meaningful inferences from plasma Aß42/40 concentrations. It has been suggested that plasma tau phosphorylated at threonine 181 (p‐tau181) and plasma glial fibrillary acidic protein (GFAP) gradually increase with amyloid and tangle deposition.^7^, ^8^ The combination of decreasing Aß42/40 and increasing p‐tau181 and GFAP therefore seems promising for the identification and prediction of AD pathology even in early stages such as subjective cognitive decline (SCD) or mild cognitive impairment (MCI). In addition, plasma neurofilament light chain (NfL) has been shown to increase with worsening AD pathology.^9^ However, NfL levels need to be interpreted cautiously due to their strong association with age and other neurological diseases.^10^ While studies have shown promising associations between AD‐related plasma biomarkers and PET measurements,^11^ there are suggestions that plasma Aβ and p‐tau may not directly reflect cerebral amyloid and p‐tau, respectively.^12^

It is therefore of interest to further explore the association between AD‐related plasma biomarkers and other neuroimaging modalities. Structural magnetic resonance (MR) imaging (MRI), functional MRI (fMRI), and MR spectroscopy (MRS) offer detailed insights into brain changes in AD. Neurodegeneration due to AD measured by MRI and connectivity measured by fMRI were shown to be associated with AD‐related plasma biomarkers.^13^, ^14^, ^15^, ^16^ Although structural and fMRI do not directly reflect amyloid and tau pathology, distinct distribution patterns of amyloid and tau deposition can be taken into consideration. In the early stages of AD, amyloid plaques predominantly accumulate in frontal and parietal areas^17^ while tau depositions on the other hand start accumulating in the inferior and lateral temporal lobes.^18^ Additionally, MRS can be used to examine biochemical changes in AD.^19^, ^20^ Myo‐inositol is a glial metabolite, primarily found in astrocytes.^20^ Myo‐inositol measured by MRS is expected to increase with glial proliferation and activation, reflecting neuroinflammatory processes related to amyloid pathology.^20^, ^21^, ^22^ N‐acetylaspartate (NAA) is a neuron‐specific metabolite primarily found in the neuronal cell body, axons, and dendrites.^20^ In MRS studies, it is widely used as a marker of neuronal integrity, with concentrations typically higher in gray matter regions and declining with age, potentially reflecting reduced neuronal density or function.^23^ Studies have linked myo‐inositol increase and NAA reduction to AD‐related brain pathology^21^, ^22^, ^24^, ^25^ and cognitive decline.^21^, ^26^ However, the association between MRS data and AD‐related plasma biomarkers remains to be explored.

This study aimed to contribute to the understanding of the association between brain changes assessed through 7 Tesla (7T) MRI and MRS and alterations in the plasma biomarkers Aß42/40, p‐tau181, GFAP, and NfL. Magnetic field strengths of 7T or more provide superior spatial resolution and an improved signal‐to‐noise ratio, resulting in more robust and reliable results.^20^, ^27^ Using the data of a cohort including cognitively healthy (HC) older adults, and individuals with SCD, MCI, or AD, we hypothesized that (1) MRI‐derived markers of neurodegeneration and connectivity predominantly in areas of early amyloid (frontal/parietal) and tau (medial temporal lobe) accumulation are reflected by changes of plasma Aß42/40 and p‐tau181, respectively; and (2) MRS‐derived biochemical brain changes related to neuroinflammation (myo‐inositol) and neuronal integrity (NAA) are reflected by changes of plasma GFAP and NfL, respectively. Associations between cognition and plasma biomarkers were assessed for comparability.

## 2 METHODS

### 2.1 Study design and participants

Data were acquired in the projects 15HLT04 NeuroMET^28^ and 18HLT09 NeuroMET2,^29^ which both aimed to improve diagnosis and management of neurodegenerative diseases using high‐quality and standardized measurement methods. Participants (*n* = 127) between 55 and 84 years of age were tested repeatedly from visit 1 to up to four follow‐up visits as indicated in Table 1. Participants were stratified into the groups HC, SCD, MCI, and dementia due to suspected AD by an experienced team of neurologists, neuropsychologists, and researchers, based on neuropsychological test results and clinical evaluation including CSF results when available (HC and SCD *n* = 0, MCI *n* = 13, AD *n* = 21). Exclusion criteria comprised history of drug/alcohol abuse or eating disorders, and severe or untreated medical, neurological, or psychiatric diseases which could potentially interfere with cognition. The main reasons for dropping out before the end of the project period were lack of ability to consent due to advanced AD (*n* = 18), lack of motivation (*n* = 29), other severe diseases (*n* = 9), and death (*n* = 2). All visits comprised a standardized medical interview, neurological examination, and extensive neuropsychological testing. At visit 1 and some follow‐up visits, participants underwent blood collection, 7T structural and fMRI, and MRS. All participants were White and native German speakers and gave written informed consent before participation in the study. The study was approved by the ethics committee of the Charité university hospital (EA1/197/16 and EA2/121/19) and was conducted in accordance with the Declaration of Helsinki.

**RESEARCH IN CONTEXT**

1. **Systematic review**: Traditional sources for review of literature and meeting abstracts were used. While there is a vast amount of literature on plasma biomarkers in Alzheimer's disease, only a few address their relationship to changes measured by magnetic resonance (MR)‐based markers of neurodegeneration, connectivity, and neuroinflammation. These relevant references are appropriately cited.
2. **Interpretation**: Our results suggest that plasma biomarkers, particularly tau phosphorylated at threonine 181 and glial fibrillary acidic protein but not amyloid beta 42/40 are associated with 7T MR‐derived changes of neurodegeneration, connectivity, and neuroinflammation. Largest effects were found for areas related to amyloid pathology, that is, frontal and parietal regions (parietal cortical thickness, connectivity of the salience network), or neuroinflammation (MR spectroscopy myo‐inositol). This contributes to previous literature suggesting that plasma biomarkers might not exclusively be related to their direct counterparts of cerebral deposition.
3. **Future directions**: The article proposes a further critical examination of the relationship between plasma biomarkers and brain changes on a regional level.

**TABLE 1 alz14318-tbl-0001:** Observations grouped by diagnosis and visit.

	HC	SCD	MCI	AD
**Visit 1**				
*n*	35	35	30	27
**Visit 2**				
*n*	27	33	17	25
Time [years]	1.0 (0.0)	1.0 (0.1)	1.0 (0.1)	1.1 (0.2)
**Visit 3**				
*n*	20	11	5	8
Time [years]	3.0 (0.2)	3.0 (0.4)	3.0 (0.2)	2.9 (0.4)
**Visit 4**				
*n*	16	8	3	4
Time [years]	4.1 (0.2)	4.2 (0.1)	4.1 (0.2)	3.9 (0.2)
**Visit 5**				
*n*	5	3	0	1
Time [years]	5.2 (0.1)	5.2 (0.2)		5.1 (0.0)

*Note*: The time difference from visit 1 is presented as mean (SD). Further statistical models might contain fewer observations according to data availability.

Abbreviations: AD, Alzheimer's disease; HC, healthy control; MCI, mild cognitive impairment; SCD, subjective cognitive decline; SD, standard deviation.

### 2.2 AD‐related plasma biomarkers

The plasma biomarkers p‐tau181, Aß42, Aß40, NfL, and GFAP were measured in one batch on the Simoa HD‐X machine with a 4× onboard dilution, using the commercially available kits NEUROLOGY 4‐PLEX E (Aß40, Aß42, GFAP, NfL, #103670) and p‐tau181 advantage kit V2 (#103714) at the Neurochemistry[Table alz14318-tbl-0001] Lab, Amsterdam. Measurements of Aß40, Aß42, GFAP, and NfL were performed in singlicate, p‐tau181 measurements were performed in duplicate and showed an average coefficient of variation% of 5.1 (range: 0.1%–19.2%) between replicates. Inter‐assay quality was monitored using two in‐house–generated pools of plasma samples. To ensure the quality of the longitudinal measurements, blood samples were all analyzed in one batch. Participants were fasting before sample collection.

### 2.3 Cognition

Memory ability was assessed by the recently developed NeuroMET Memory Metric (NMM). The NMM is a composite metric that comprises 57 dichotomous items in a bank of items carefully selected from legacy short‐term memory tests linking language‐ and cultural‐free items (blocks, digits) to more complex word‐recalling items.^30^ It was developed as a part of the NeuroMET projects and has been demonstrated to have superior accuracy compared to previous memory metrics without jeopardizing validity.^31^ Executive function was assessed by a composite score defined by the mean of the *z* transformed values for semantic fluency (animals), phonemic fluency (s‐words, according to Morris et al.^32^), completion time for the Stroop test C,^33^ completion time for the Trail‐Making Test Part B (TMT‐B,^34^), and digit symbol substitution (Wechsler Adult Intelligence Scale,^35^). A maximum of two missing items were allowed for the composite score of executive function.

### 2.4 Structural MRI

Using a 7T whole‐body scanner (Magnetom 7T, Siemens Healthineers), high‐resolution structural cerebral images were acquired using a three‐dimensional T1‐weighted magnetization‐prepared 2 rapid acquisition gradient echoe sequence (MP2RAGE,^36^) with a denoised reconstruction,^37^ using the following parameters: repetition time/echo time (TR/TE) = 5000 ms/2.51 ms, TI1/TI2 = 900 ms/2700 ms, bandwidth = 250 Hz/Pixel, α1/α2 = 7°/5°, 0.75 mm isotropic resolution, field of view (FoV) 240 × 240 × 180 mm^3^, generalized autocalibrating partial parallel acquisition (GRAPPA) acceleration factor 2, 32 reference lines, 11 minutes 17 seconds total acquisition time.

**FIGURE 1:**
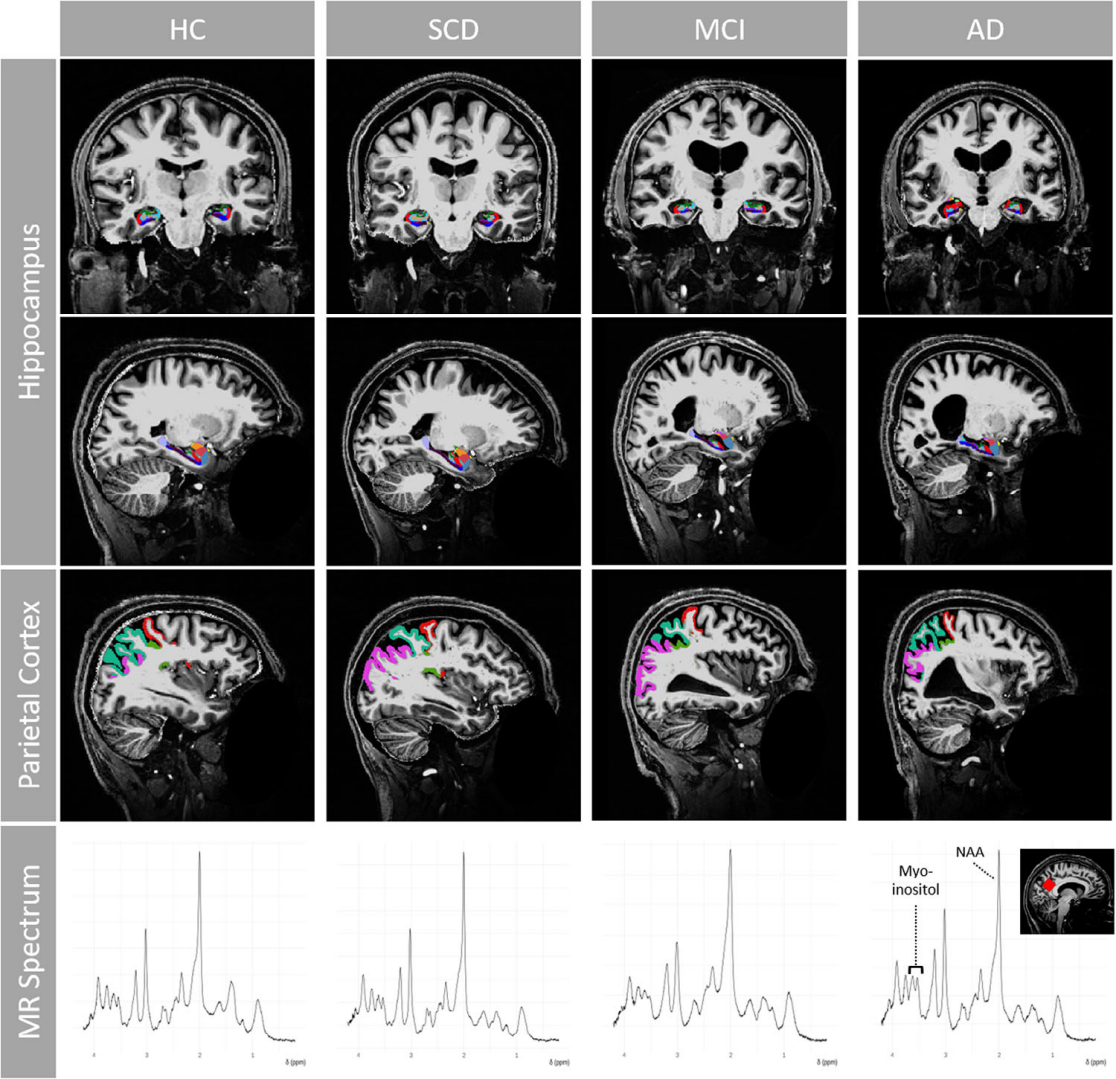
Structural MR images and spectra from participants from the clinically defined groups HC (age = 61), SCD (age = 62), MCI (age = 70), and AD (age = 66) using a 7T whole‐body scanner (Magnetom 7T, Siemens Healthineers). Structural data were acquired by T1‐weighted MP2RAGE and segmented by FreeSurfer 7.1.1. For this study, we selected the thickness of the ROIs of the parietal lobe (superior and inferior parietal, supramarginal, postcentral, precuneus, and PCC) and the volume of the hippocampus (extracted from the subfield pipeline, Figure 1 shows the left hippocampus). MRS data on myo‐inositol and NAA concentration were acquired in a 20 × 20 × 20 mm^3^ MRS volume‐of‐interest positioned in the sagittal PCC/precuneus region (see the red square in the inlet) using the SPECIAL sequence and the software LCModel v6.3. AD, Alzheimer's disease; HC, healthy control; MCI, mild cognitive impairment; MP2RAGE, magnetization‐prepared 2 rapid acquisition gradient echo sequence; MR, magnetic resonance; MRS, magnetic resonance spectroscopy; NAA, N‐acetylaspartate; PCC, posterior cingulate cortex; ROIs, regions of interest; SCD, subjective cognitive decline; SPECIAL, spin echo full intensity acquired localization.

The pipeline for (pre‐)processing of the images was published in a previous publication^38^ and the code is available on GitHub.^39^ Briefly, we used the open‐source FreeSurfer 7.1.1 image analysis suite (Martinos Center for Biomedical Imaging, Massachusetts General Hospital, http://surfer.nmr.mgh.harvard.edu/,^40^) to segment the acquired T1 weighted images. For participants who had performed multiple visits, measurements were further processed using the longitudinal FreeSurfer pipeline. For this study, we selected the thickness of the regions of interest (ROIs) of the parietal lobe (superior and inferior parietal, supramarginal, postcentral, precuneus, and posterior cingulate cortex [PCC]) and the volume of the hippocampus (extracted from the subfield pipeline). Examples of segmentation results are shown in Figure 1. Segmentations were visually inspected and excluded when quality was not sufficient. Parietal thickness was defined as the weighted average of the thickness of the mentioned ROIs belonging to the parietal lobe, using the surface as a scaling factor with the formula: parietal thickness = Σ_ROI_ (ROI thickness × ROI area)/Σ_ROI_ (ROI area). Hippocampus volume was adjusted to the individual total intracranial volumes (TIV) and normalized to the mean TIV of the HC group (mean TIV) according to Voevodskaya et al.^41^ using the formula: adjusted volume = raw volume − *b* × (TIV—mean TIV), where the coefficient *b* represents the slope of the regression between the hippocampus volume and the TIV of healthy participants. For the analyses in this study, left and right measurements were summed to provide one single value for each of the two structural MR‐derived measures.

### 2.5 MRI

Blood oxygen level–dependent (BOLD) data were acquired using a multiband multislice gradient echo–echo planar imaging (EPI) sequence^42^, ^43^: TR/TE = 2200 ms/28.2 ms, echo spacing = 0.97 ms, 1.5 mm isotropic resolution, FoV = 240 × 240 × 120 mm^3^, flip angle = 66°, GRAPPA acceleration factor 2, 32 reference lines, multiband factor 4, 300 measurements. The axial slices were oriented parallel to the imaginary line from the commissura anterior to the commissura posterior, and subjects were instructed to lay still with their eyes closed and to let their mind wander. An additional B0 map (TR/TE1/TE2 = 500 ms/5 ms/7.02 ms, 3 mm isotropic resolution) using the same B0 shim settings and orientation was acquired for distortion correction. Data were preprocessed with fMRIPrep v20.1.1,^44^ denoised using NiLearn v0.10.1.^45^ The code for our pipeline is available on GitHub.^46^

**FIGURE 2:**
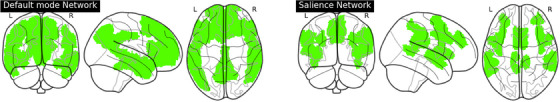
Glass brains to visualize the default mode network (left) and salience network (right). ROIs were defined using the 7 networks, 400 ROIs Schaefer Atlas.^34^ ^.^ ROIs, regions of interest.

The functional connectivities were extracted using the Schaefer Atlas 2018 (7 Networks, 400 ROIs,^47^) using Pearson's correlation as a connectivity measure. The connectivity matrices were extracted as *z* scores using NiLearn. Connectivity values of ≤ 0 were excluded and the lower triangular matrix was selected for each subject. From the matrix, functional connectivity values were extracted for the ROIs of the default mode network (DMN) and the salience network (Sal; Figure 2) and were averaged for each of the two networks. Data from two observations were excluded because of insufficient data quality (i.e., failed co‐registration, suspicious connectivity matrix).

### 2.6 MRS

The 20 × 20 × 20 mm^3^ MRS volume of interest was positioned in the sagittal PCC/precuneus region (see inlet in Figure 1). Localized radio frequency calibration and second order B_0_ shimming^48^, ^49^ were performed before single‐voxel spectra were acquired using the spin echo full intensity acquired localization (SPECIAL) sequence^50^ with interleaved outer volume suppression and variable power and optimized relaxations delays (VAPOR)^51^ water suppression (TR/TE = 6500 ms/9.0 ms, number of acquisitions = 64, number of sample points = 2048, acquisition duration = 512 ms, excitation bandwidth = 4000 Hz, water suppression bandwidth = 60 Hz). Additionally, a non–water‐suppressed spectrum was acquired for reference (number of acquisitions = 4; otherwise identical parameters).

The MRS raw data were preprocessed using a MATLAB‐based in‐house developed reconstruction algorithm. At first, each odd acquisition was averaged with the next even acquisition, to obtain a fully localized spectrum. Subsequently, a weighted, phase‐corrected coil combination was performed, before the remaining fully localized signals were frequency aligned to the NAA methyl peak at 2.00 ppm and finally averaged.

The preprocessed spectra were analyzed using LCModel v6.3^52^ with a simulated basis set, generated in VeSPA v0.9.3.^53^ Example spectra are shown in Figure 1. After absolute quantification, the water‐scaled measurements for myo‐inositol and NAA comprised partial volume correction.^54^ Additionally, Cramér‐Rao lower bounds (CRLBs,^55^), indicators for individual measurement uncertainty, were extracted from the LCModel output files.

### 2.7 Statistics

Statistical analyses were performed using R v4.0.2,^56^ using the lme4,^57^ emmeans,^58^ and tidyverse^59^ packages. The full reproducible code is available elsewhere.^60^ No adjustment for multiple testing was applied except for Tukey post hoc tests after group comparisons. Therefore, *P* values must be interpreted with caution. Interpretation of results is primarily based on effect estimates and corresponding 95% confidence intervals (CIs). All models with MRS concentrations (myo‐inositol and NAA) were weighted based on CRLBs as suggested in previous work.^61^

Group differences were tested using the Kruskal–Wallis rank‐sum test (continuous demographics), Pearson chi‐squared test (dichotomous demographics), multiple regression adjusted for age (MR‐derived biomarkers), or adjusted for age and years of education (cognition).

Cross‐sectional group differences (Tukey adjusted) and group‐wise longitudinal course of plasma biomarkers and outcome variables were explored using linear mixed models adjusted for age (model 1). Cross‐sectional and longitudinal associations between plasma biomarkers and outcome variables (MR‐derived measurements or cognition) were examined similarly by linear mixed models, across the total study population adjusting for age, sex, and education (model 2). Models are explained in detail in the supporting information (Appendix S1).

To compare the associations between plasma biomarkers and the AD‐related outcome variables, we transformed the concentration of plasma biomarkers and outcome (MR‐derived measurements or cognition) to *z* scores based on the mean and standard deviation (SD) of the HC group. Therefore, effects are reported as standardized ß coefficients (std. ß). For ease of interpretation of interaction effects in model 2, we report yearly changes of the outcome for three discrete yearly changes of plasma biomarkers: (1) stable HC mean (“normal stable”), (2) stable HC mean – 1 SD (for Aß42/40) or HC mean + 1 SD (for p‐tau181, GFAP, NfL; “abnormal stable”), and (3) change from normal to abnormal (“normal ⇒ abnormal”). Yearly changes of the outcome measurements of > ± 0.1 SD toward the pathological direction were considered relevant and discrete values are listed in Table S4 in supporting information.

## 3 RESULTS

### 3.1 Characteristics of the study sample

Group‐wise number of included observations per visit for the 127 participants is presented in Table 1. Table 2 presents the participants’ characteristics and measurements at visit 1. Participants of the AD group were on average older than participants of the other groups and there were fewer female participants in the MCI group than in the remaining groups. There were more carriers of apolipoprotein E (*APOE*) ε4 in the MCI and AD group compared to the other groups. Cognitive functions measured by the Mini‐Mental State Examination,^62^ NMM, and executive function were gradually lower in the AD group. On average, participants from the SCD group showed values similar to, or better than, the HC group. To enhance the comprehensibility of the dataset, a correlation matrix was included in the supporting information (Figure S2), showing how age, education, cognition, and imaging biomarkers related to each other.

**TABLE 2:** Participants’ characteristics at visit 1.

		Diagnosis at visit 1				
	Total	HC	SCD	MCI	AD	
	*N* = 127	*N* = 35	*N* = 35	*N* = 30	*N* = 27	*p*‐value
**Demographics**						
Age [years]	72 (7)	71 (8)	69 (7)	71 (6)	75 (6)	**0.005**
Female	63 (50%)	18 (51%)	22 (63%)	8 (27%)	15 (56%)	**0.027**
Education [years]	15 (3)	15 (3)	16 (2)	15 (3)	14 (3)	0.212
*APOE* ε4 carrier	52 (41%)	9 (26%)	11 (31%)	17 (57%)	15 (56%)	**0.018**
MMSE	27.26 (3.46)	28.97 (1.07)	29.00 (0.97)	27.07 (2.16)	22.68 (4.74)	**<0.001**
*N* missing	2	0	0	0	2	
**Cognition**						
NMM	0.00 (1.05)	0.60 (0.51)	0.65 (0.80)	−0.33 (0.65)	−1.25 (0.92)	**<0.001**
Executive function	−0.64 (1.24)	0.01 (0.74)	0.21 (0.58)	−1.16 (1.06)	−2.04 (1.11)	**<0.001**
*N* missing	1	0	0	0	1	
**Structural MRI**						
Hippocampus [mm^3^]	2652 (439)	2875 (306)	2909 (336)	2512 (382)	2187 (315)	**<0.001**
*N* missing	17	3	6	5	3	
Parietal cortical thickness [mm]	2.20 (0.13)	2.22 (0.10)	2.23 (0.10)	2.16 (0.10)	2.15 (0.20)	**0.031**
*N* missing	18	3	7	4	4	
**fMRI**						
DMN connectivity	0.33 (0.05)	0.33 (0.05)	0.34 (0.04)	0.32 (0.04)	0.32 (0.05)	0.093
*N* missing	14	3	5	2	4	
Sal connectivity	0.35 (0.05)	0.35 (0.05)	0.38 (0.04)	0.35 (0.06)	0.32 (0.05)	**<0.001**
*N* missing	14	3	5	2	4	
**MRS**						
Myo‐inositol [mmol/L]	7.57 (1.44)	7.23 (1.01)	6.85 (1.09)	7.64 (1.33)	8.75 (1.68)	**< 0.001**
*N* missing	11	3	5	2	1	
NAA [mmol/L]	15.12 (1.76)	15.57 (1.66)	14.89 (1.42)	15.01 (2.22)	14.94 (1.66)	0.334
*N* missing	11	3	5	2	1	
**Plasma biomarkers**						
Aß42/40 [pg/mL]	0.065 (0.011)	0.067 (0.012)	0.067 (0.013)	0.063 (0.010)	0.060 (0.009)	**0.043**
p‐tau181 [pg/mL]	2.47 (1.47)	2.08 (1.01)	1.81 (0.62)	2.63 (1.39)	3.68 (2.03)	**<0.001**
GFAP [pg/mL]	148 (90)	107 (47)	120 (48)	144 (77)	241 (119)	**<0.001**
NfL [pg/mL]	25 (18)	19 (9)	20 (7)	23 (10)	40 (31)	**<0.001**

*Note*: Mean (SD) or *N* (%) are shown for the total study population and the individual groups. Group differences were tested using the Kruskal–Wallis rank‐sum test (continuous demographics), Pearson chi‐squared test (dichotomous demographics), multiple regression adjusted for age (MR‐based and plasma biomarkers), or adjusted for age and education (cognition). The statistical models for MRS data (myo‐inositol and NAA) were additionally weighted based on an estimation of measurement uncertainties of MRS concentrations (Cramér‐Rao lower bounds). Group differences with *p*‐values < 0.05 are presented in bold. *APOE* ε4 carriers were identified using TaqMan assays.

Abbreviations: Aß, amyloid beta; AD, Alzheimer's disease; *APOE*, apolipoprotein E; DMN, default mode network; fMRI, functional magnetic resonance imaging; GFAP, glial fibrillary acidic protein; HC, healthy control; MCI, mild cognitive impairment; MMSE, Mini‐Mental State Examination; MR, magnetic resonance; MRI, magnetic resonance imaging; MRS, magnetic resonance spectroscopy; NAA, N‐acetylaspartic acid; NfL, neurofilament light chain; NMM, NeuroMET Memory Metric; p‐tau181, tau phosphorylated at threonine 181; Sal, salience network; SCD, subjective cognitive decline; SD, standard deviation.

### 3.2 Plasma biomarkers: cross‐sectional concentrations and longitudinal trajectories

Concentrations of plasma Aß42/40, p‐tau181, GFAP, and NfL at visit 1 can be found alongside the demographics in Table 2, and visualized in Figure S1 in supporting information. At visit 1, the AD group showed similar concentrations as the HC group for Aß42/40 (model 1, Tukey adjusted mean difference [95% CI] = −0.007 pg/mL [−0.014; 0.001], *P* = 0.102), but higher p‐tau181 (1.39 pg/mL [0.56; 2.22], *P* < 0.001), GFAP (120 pg/mL [71; 169], *P* < 0.001), and NfL (19 pg/mL [8; 29], *P* < 0.001). Neither the SCD nor MCI groups showed significant differences in the concentrations of any of the plasma biomarkers compared to HC (*P* > 0.150).

**FIGURE 3:**
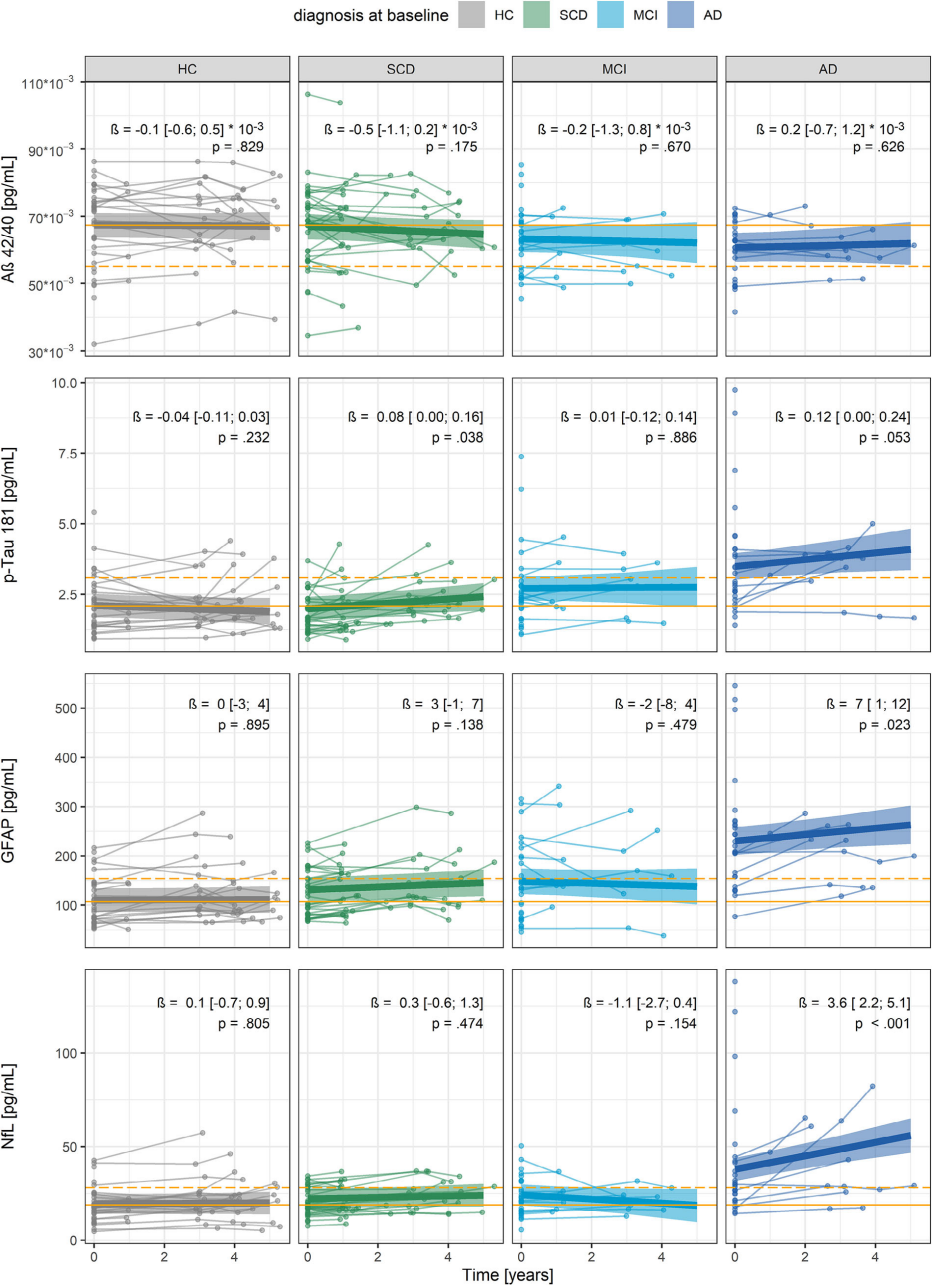
Description of longitudinal concentration changes of plasma Aß42/Aß40, p‐tau181, GFAP, and NfL. In the SCD group, only plasma p‐tau181 showed a relevant yearly increase. The AD group, however, showed substantially increasing concentrations of p‐tau181, GFAP, and NfL. Concentrations were measured at visit 1 (*n* = 127), and when available at follow‐ups after 1 year (*n* = 32), 3 years (*n* = 44), 4 years (*n* = 31), and 5 years (*n* = 9). The thin lines link individual concentrations at each available visit. The linear fits are presented with 95% CI (shaded areas) estimated by linear mixed models adjusted for age. For later interpretation of the models, orange lines represent the mean of the HC group at visit 1 (continuous line) and 1 SD toward the pathological direction (dashed line). Aß, amyloid beta; AD, Alzheimer's disease; CI, confidence interval; GFAP, glial fibrillary acidic protein; HC, healthy control; MCI, mild cognitive impairment; NfL, neurofilament light chain; p‐tau181, tau phosphorylated at threonine 181; SCD, subjective cognitive decline; SD, standard deviation.

The longitudinal trajectory of the plasma biomarkers is shown in Figure 3. The steepest decrease of plasma Aß42/40 was found in the SCD group (model 1); however, the effect was small, with large uncertainties (Figure 3). Overall, only plasma p‐tau181 showed a substantial yearly increase in the SCD group. The AD group, but not the MCI group, showed a relevant increase in concentrations of p‐tau181, GFAP, and NfL.

### 3.3 7T structural and fMRI and MRS: cross‐sectional and longitudinal measurements

MR‐derived measurements and cognition at visit 1 can be found alongside the demographics in Table 2. At visit 1, the AD group showed smaller hippocampus volumes (model 1, Tukey adjusted mean difference [95% CI] = −614 m^3^ [−391; −837], *P* < 0.001), and higher myo‐inositol (1.41 [0.51; 2.30], *P* < 0.001), but similar parietal cortical thickness (−0.06 mm [−0.15; 0.02], *P* = 0.231), DMN connectivity (0.00 [−0.04; 0.03], *P* = 0.987), Sal connectivity (−0.03 [−0.06; 0.01], *P* = 0.174), and NAA (−0.60, [−1.77; 0.58], *P* = 0.552) compared to the HC group. The SCD group showed similar or better MR‐derived values compared to the HC group (Table 2).

**FIGURE 4:**
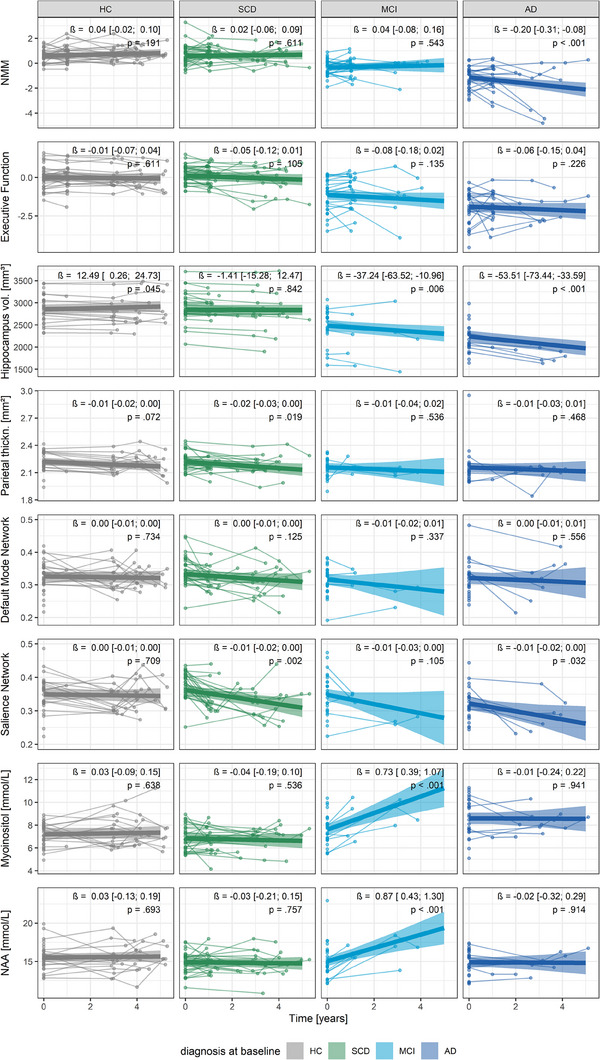
Description of longitudinal changes of cognition, structural and fMRI, and MRS. The thin lines link individual measurements in their individual unit at each available visit. The linear fits are presented with 95% CI (shaded areas) estimated by linear mixed models adjusted for age. Models including the MRS parameters myo‐inositol and NAA were additionally weighted based on a measure of uncertainty (CRLB). Although on average, the HC, SCD, and MCI groups showed stable cognition over time, there were changes in hippocampus volume, parietal cortical thickness, salience network connectivity, myo‐inositol, and NAA. Besides a substantial cognitive decline in the AD group, individuals showed a decrease in hippocampus volume and salience network connectivity. Due to the small sample size for longitudinal data and the grouping being based on clinical and not pathological diagnostics, further models of associations between MR‐derived measurements and plasma biomarkers did not include groups. AD, Alzheimer's disease; CI, confidence interval; CRLB, Cramér‐Rao lower bound; fMRI, functional magnetic resonance imaging; HC, healthy control; MCI, mild cognitive impairment; MRI, magnetic resonance imaging; MRS, magnetic resonance spectroscopy; NAA, N‐acetylaspartic acid; NMM, NeuroMET Memory Metric; SCD, subjective cognitive decline; thickn., thickness; vol., volume.

The longitudinal trajectories of measurements of cognition, structural and fMRI, and MRS are shown in Figure 4. While MR‐derived measurements generally tended to worsen toward the AD group, the amount of longitudinal data for both MCI and AD groups was limited. To mitigate potential biases due to small sample sizes and the mismatch between clinical diagnosis and underlying brain pathology, further analyses of the relationships between MR‐derived measurements and plasma biomarkers were conducted independently of the clinical diagnoses (model 2).

### 3.4 Cross‐sectional association between MR‐derived measurements and plasma biomarkers

**FIGURE 5:**
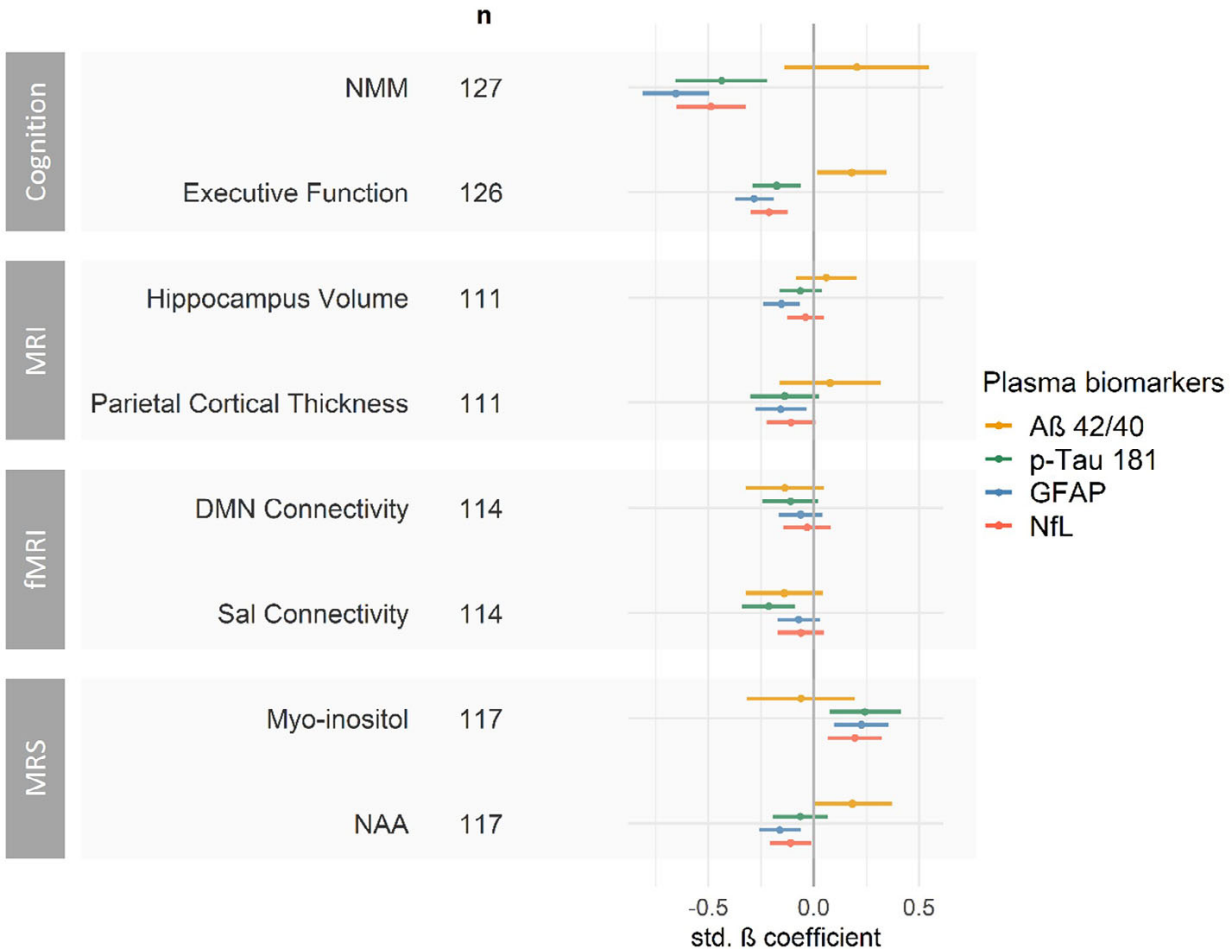
Cross‐sectional associations between concentrations of plasma Aß42/40, p‐tau181, GFAP, and NfL and *z* transformed parameters of cognition, structural and fMRI, and MRS (at visit 1). Largest effect sizes were consistently found for either p‐tau181 or GFAP. Effects for Aß42/40 were smaller with large uncertainties. Effects for NMM, that is, memory ability, were considerably larger than for any of the MR‐based parameters. Depending on data availability, observations of a maximum of 127 participants were included. The effects are presented with 95% CI (horizontal bars) estimated by linear mixed models adjusted for age, sex, and education. Models including the MRS parameters myo‐inositol and NAA were additionally weighted based on a measure of uncertainty (CRLB). Connectivity measures for the DMN and Sal were acquired at resting state. Aß, amyloid beta; CI, confidence interval; CRLB, Cramér‐Rao lower bound; DMN, default mode network; fMRI, functional magnetic resonance imaging; GFAP, glial fibrillary acidic protein; MR, magnetic resonance; MRI, magnetic resonance imaging; MRS, magnetic resonance spectroscopy; NAA, N‐acetylaspartic acid; NfL, neurofilament light chain; NMM, NeuroMET Memory Metric; p‐tau181, tau phosphorylated at threonine 181; Sal, salience network.

Using linear mixed models (model 2), we found multiple relevant associations between plasma biomarkers and MR‐derived measurements at visit 1, as indicated in Figure 5 and Table S2 in supporting information. However, contrary to our first hypothesis, neurodegeneration and connectivity in areas of early amyloid (parietal cortical thickness, Sal connectivity) and tau accumulation (hippocampus volume, DMN connectivity) were not associated with plasma Aß42/40 or p‐tau181, respectively. Instead, we observed that smaller hippocampus volume and parietal cortical thickness were only associated with plasma GFAP. Connectivity of the DMN showed no association with any of the plasma biomarkers, while Sal connectivity was associated with p‐tau181. Further, in line with our second hypothesis, higher myo‐inositol and lower NAA were associated with higher levels of plasma GFAP and NfL, respectively. However, higher myo‐inositol was also associated with higher p‐tau181 and NfL, and lower NAA was also associated with higher GFAP.

In summary, of all models revealing substantial associations between plasma biomarkers and MR‐derived measurements, the largest effect sizes were consistently found for either plasma p‐tau181 (Sal connectivity, myo‐inositol) or GFAP (hippocampus volume, parietal cortical thickness, NAA). Notably, associations between plasma Aß42/40 and MR‐derived measurements showed large 95% CIs, indicating higher uncertainty in its relationships to all MR‐derived measurements compared to p‐tau181, GFAP, and NfL. However, without exception, associations to plasma biomarkers were by far stronger for memory ability measured by the NMM than for any of the MR‐derived measurements. Effect sizes for the association to the NMM reached ≈ 2‐fold (p‐tau181, NfL) or 4‐fold (GFAP) greater magnitudes than for the associations to any MR‐derived measurement.

### 3.5 Longitudinal association between plasma biomarkers and MR‐derived measurements

**FIGURE 6:**
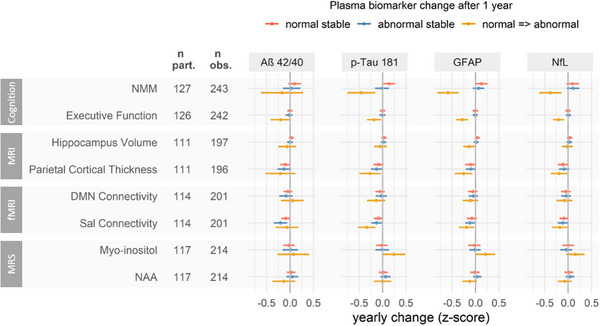
Yearly change of cognitive, structural, and fMRI, and MRS parameters depending on changes of plasma Aß42/40, p‐tau181, GFAP, and NfL. To allow for comparison, all parameters were z‐transformed. Yearly changes were estimated for discrete changes of plasma biomarker concentrations after 1 year: stable concentrations of the mean of the HC group (normal stable, blue), change of concentration from the mean of the HC group to 1 SD toward the pathological direction as indicated in Figure 1 (normal ⇒ abnormal, red), stable concentrations of 1 SD toward the pathological direction (abnormal stable, blue). Depending on data availability, a maximum of 127 participants with 243 observations were included. The effects are presented with 95% CI estimated by linear mixed models adjusted for age, sex, and education. Models including the MRS markers myo‐inositol and NAA were additionally weighted based on a measure of uncertainty (CRLB). Aß, amyloid beta; CI, confidence interval; CRLB, Cramér‐Rao lower bound; DMN, Default mode network; fMRI, functional magnetic resonance imaging; GFAP, glial fibrillary acidic protein; HC, healthy control; MRI, magnetic resonance imaging; MRS, magnetic resonance spectroscopy; NAA, N‐acetylaspartic acid; NfL, neurofilament light chain; NMM, NeuroMET Memory Metric; obs., observations; part., participants; p‐tau181, tau phosphorylated at threonine 181; Sal, salience network; SD, standard deviation.

We further explored whether MR‐derived measurements were reflected by plasma biomarker changes over time. Interaction effects (plasma biomarkers × time) are reported in Table S3 in supporting information. To ease interpretation of interaction effects, we report estimates for yearly changes of cognition or MR‐derived measurements for three defined scenarios of plasma biomarker change after 1 year: (1) participants who present stable concentrations similar to the HC mean (“normal stable”), (2) participants who present stable concentrations similar to HC mean ± 1 SD toward the pathological direction (“abnormal stable”), and (3) participants whose concentration change from the HC mean to ± 1 SD toward the pathological direction (“normal ⇒ abnormal”). Figure 3 visualizes the concentrations that were considered to be normal (HC mean) and abnormal (± 1 SD). The results of all estimations are shown in Figure 6 with accurate values presented in Table S4.

We found several relevant associations (yearly changes > 0.1 SD) for a plasma biomarker change normal ⇒ abnormal, with strongest effects between yearly changes of parietal cortical thickness, connectivity of Sal, and myo‐inositol and plasma p‐tau181 or GFAP as reported in the following sections. However, yearly changes of NMM, that is, memory ability, were again larger than changes of any of the MR‐derived measurements: Declining memory ability was associated with increased p‐tau181 (−0.45 SD [−0.75; −0.16], *P* < 0.001), GFAP (−0.59 SD [−0.82; −0.35], *P* < 0.001), and NfL (−0.38 SD [−0.62; −0.15], *P* < 0.001), but not Aß42/40 (−0.17 SD [−0.62; 0.28], *P* = 0.759).

#### 3.5.1 Neurodegeneration and decreasing connectivity reflected by increased plasma p‐tau181, GFAP, and NfL

Yearly changes in parietal cortical thickness and Sal connectivity were not associated with normal ⇒ abnormal plasma Aß42/40 (parietal cortical thickness −0.21 SD [−0.52; 0.11], *P* = 0.337, Sal connectivity −0.06 SD [−0.30; 0.18], *P* = 0.902), and changes in hippocampus volume and DMN connectivity were not associated with normal ⇒ abnormal plasma p‐tau181 (hippocampus volume −0.05 SD [−0.18; 0.09], *P* = 0.786). Thus, neurodegeneration and decreasing connectivity in frontal/parietal or medial temporal lobe regions were not reflected by changes in plasma Aß42/40 or p‐tau181, respectively.

Although not associated with the hypothesized respective plasma biomarkers, neurodegeneration and decreasing connectivity were associated with changes in plasma p‐tau181, GFAP, and NfL. Specifically, decreasing hippocampus volume was associated with normal ⇒ abnormal plasma GFAP (−0.14 SD [−0.26; −0.01], *P* = 0.023), and decreasing parietal cortical thickness was associated with normal ⇒ abnormal plasma p‐tau181 (−0.27 SD [−0.50; −0.04], *P* = 0.014), GFAP (−0.25 SD [−0.44; −0.07], *P* = 0.003) and NfL (−0.19 SD [−0.37; −0.02], *P* = 0.027). Further, decreasing Sal connectivity was associated with normal ⇒ abnormal plasma p‐tau181 (−0.34 SD [−0.53; −0.16], *P* < 0.001), GFAP (−0.19 SD [−0.35; −0.03], *P* = 0.014), and NfL (−0.18 SD [−0.35; −0.01], *P* = 0.030). Consistent with the cross‐sectional analysis, changes in DMN connectivity were not associated with any of the plasma biomarkers, and normal ⇒ abnormal plasma Aß42/40 was not associated with changes in any of the MR‐derived measurements.

#### 3.5.2 Neuroinflammation indicated by increasing MRS myo‐inositol reflected by increased plasma GFAP

Increasing myo‐inositol was associated with normal ⇒ abnormal plasma GFAP (0.22 SD [−0.01; 0.42], *P* = 0.036), and potentially with normal ⇒ abnormal plasma p‐tau181 (0.24 SD [−0.01; 0.48], *P* = 0.063), although not reaching significance. However neuronal integrity estimated by decreasing MRS NAA was not associated with plasma NfL (−0.07 SD [−0.22; 0.08], *P* = 0.613) or any other plasma biomarker.

## 4 DISCUSSION

In this observational study, we explored the associations between measurements of (1) 7T MRI‐derived neurodegeneration and connectivity of areas of early amyloid or tau deposition, and plasma Aß42/40 and p‐tau181, respectively, and (2) neuroinflammation and neuronal integrity indicated by 7T MRS and plasma GFAP and NfL, respectively. Neurodegeneration and decreasing connectivity were associated with increasing plasma p‐tau181, GFAP, and NfL; however, these associations were not exclusive to the hypothesized MR‐derived measurements. Neuroinflammation assessed through MRS myo‐inositol was reflected by plasma GFAP. Despite generally showing small effect sizes, plasma NfL, along with GFAP, reflected neuronal integrity assessed through MRS NAA, but this was observed only in the cross‐sectional analysis. Notably, associations between Aß42/40 and all 7T MRI‐derived measurements were weak and presented large 95% CIs, suggesting a lack of robustness in the relationship between plasma Aß42/40 and MR‐derived brain changes. Overall, plasma p‐tau181 and GFAP showed the most robust associations with MR‐derived measurements beyond their hypothesized corresponding MR‐derived measurements.

We aimed to understand cross‐sectional and longitudinal associations of plasma biomarkers with 7T MR‐derived measurements for neurodegeneration, functional connectivity, neuroinflammation, and neuronal integrity, which typically become abnormal in AD. The magnetic field strength of 7T enhances the precision of MRI and MRS data by providing superior spatial resolution and an improved signal‐to‐noise ratio, resulting in more robust and reliable results. Contrary to our first hypothesis, neither parietal cortical thickness nor decreased connectivity of the Sal network, both of which include areas of early amyloid aggregation, were associated with plasma Aß42/40. All cross‐sectional and longitudinal associations between 7T MR‐derived measurements and plasma Aß42/40 were consistently weaker and presented larger 95% CIs than for p‐tau181 and GFAP (Figures 5 and 6), which is in line with previous studies.^3^ The lack of associations for plasma Aß42/40 might be due to plasma Aß42/40 levels reaching a plateau by the time MR‐derived measurements become abnormal, while changes in p‐tau181 and GFAP occur closer to emerging changes of MR‐derived measurements. The large 95% CIs might indicate limited accuracy for plasma Aß measurements, particularly by single molecule array, as previously observed.^63^ Alternatively, they may reflect a discordance between cerebral and plasma amyloid levels, potentially influenced by confounding peripheral factors such as body mass index, and kidney and liver function.^64^ The data of this study therefore support the use of plasma p‐tau181 and GFAP over Aß42/40 for monitoring the progression of AD.

Although decreased hippocampus volume and decreased DMN connectivity (including areas typically affected by tau depositions) were not associated with increased plasma p‐tau181 as hypothesized, we found other relevant associations between 7T MR‐derived measurements and plasma p‐tau181. Plasma GFAP, which was related to neuroinflammation indicated by MRS myo‐inositol as hypothesized, showed additional relevant associations with other 7T MR‐derived measurements. An exploratory analysis in the supporting information further indicates that higher plasma GFAP was only significantly associated with smaller hippocampus volume among individuals classified as p‐tau positive but not p‐tau negative (Figure S4, Table S4), which confirms GFAP's role in AD pathology versus normal aging. Associations between p‐tau181 or GFAP and neurodegeneration of temporal regions including the hippocampus have previously been shown to be relevant^65^ but have been usually weak.^3^, ^13^, ^66^ Longitudinal associations between hippocampus volume and plasma p‐tau181 were not substantial in a previous study.^65^ While we did not have data quantifying cerebral tau depositions, some previous studies support the theory that plasma p‐tau181 and GFAP do not specifically reflect tau aggregation or neuroinflammation, respectively: Although plasma p‐tau181 was shown to correlate with *post mortem* tangle load^1^ and cortical tau protein deposition measured by 18F‐flortaucipir PET,^67^ it was shown to be equally^1^ or even more closely^12^ associated to amyloid pathology *post mortem* or per PET, respectively. Moscoso et al.^7^ suggested that increasing plasma p‐tau181 is associated with widespread cortical Aß pathology and prospective tau aggregation (after ≈ 6 years) rather than momentary tau aggregation. Plasma GFAP, on the other hand, showed heterogeneous results, for example, being only associated with tangle but not amyloid pathology^1^ or vice versa.^68^ Plasma p‐tau181 and GFAP might therefore be less specific to changes resulting from actual tau deposition and rather represent effects of disease progression in terms of Aß pathology and subsequent neuroinflammatory processes.

Although lower NAA was associated with higher plasma NfL cross‐sectionally, decreasing NAA over time was not reflected by normal ⇒ abnormal plasma NfL, which only partially confirms our second hypothesis. MRS NAA has been reported to be decreased due to AD and is thought to be a marker of neuronal integrity.^20^ In the present dataset, however, particularly in individuals of the MCI group, there was no decrease in NAA over time as presented in Figure 4, possibly due to the limited amount of longitudinal MRS data for this group. These findings suggest that larger longitudinal samples are needed to confirm the relationship between NAA changes and plasma NfL levels in neurodegenerative diseases.

Additionally, longitudinal models showed negligible changes in the 7T MR‐derived measurements for participants with stable plasma biomarkers irrespective of the level at visit 1 (normal or abnormal). MR‐derived measurements were primarily associated with the change in plasma biomarker concentrations (normal ⇒ abnormal). Detrimental brain changes without changing plasma biomarkers might point to other causes than AD. Our results, therefore, indicate that single measurements of plasma concentrations might not be sufficient to predict brain changes and long‐term observations seem appropriate for early diagnosis, a hypothesis to be evaluated in future longitudinal studies with larger cohorts.

Ultimately, associations between MR‐derived measurements and plasma biomarkers were considerably weaker than the association between memory ability measured by the NMM and plasma biomarkers. A strong association between AD‐related plasma biomarkers and memory functions has been reported before^3^ but has not been compared to MR‐derived associations. Note that participants with normal stable concentrations of p‐tau181 showed a substantial yearly improvement in memory ability, which may be due to learning effects. For participants with abnormal, stable concentrations of plasma p‐tau181, memory ability did not improve. This suggests that initial concentrations (normal or abnormal) are relevant for predicting memory decline, but not changes in MR‐derived measurements as discussed before. Alternatively, the discrepancy in effect sizes between models of memory ability and any of the MR measurements could be due to improved accuracy of the memory measurement chosen in our study as previously reported.^31^ Another possible reason might be the difference in statistical power due to differences in the number of observations for measures of cognition versus MR data (sample sizes are reported in Figures 5 and 6). Furthermore, Figure 4 shows available data points and visualizes that particularly the MCI and AD groups lack data points for MR‐derived measurements but not cognition, possibly leading to a mismatch for the samples of the models. Consequently, we refrain from drawing any finite conclusion from the relatively larger effect sizes for memory ability compared to MR‐derived measurements and encourage further studies.

This study has limitations that should be considered. First, amyloid status by CSF or PET was not available for all individuals, most notably for HC and SCD, which are the stages in which we would expect the strongest decrease of plasma Aß42/40. Especially the SCD group might be a heterogeneous group with mixed causes for their subjective cognitive decline. Although the results of this study therefore represent a valuable setting more closely related to real life than a controlled study environment, the presented group‐wise effects (model 1) might be more pronounced in cohorts with only AD biomarker positive participants. To prevent bias, we performed the main analysis (model 2) independent of the grouping, that is, across the whole cohort. Second, changes in plasma biomarker concentrations in early stages seem to be subtle^65^, ^69^ and therefore some associations might require larger data sets to reach significance. Larger sample sizes would also allow for the exploration of plasma biomarker trajectories beyond the assumption of a linear course, and consider subtypes, for example, individuals with pronounced versus subtle changes could be examined separately, enabling personalized medicine. Nevertheless, the study aimed to optimize robustness by using high‐resolution MR data and can therefore be used to reach relevant conclusions.

## 5 CONCLUSION

Overall, our results suggest that 7T MR‐derived measurements indicative of AD progression are reflected by plasma p‐tau181 and GFAP, while effects are not limited to brain changes in areas of early tau deposition or neuroinflammation measured by MRS myo‐inositol, respectively. The relationship between brain changes and plasma biomarkers in AD appears to be complex, indicating the need for further investigation into their underlying mechanisms and clinical implications.

## CONFLICT OF INTEREST STATEMENT

Charlotte E. Teunissen has research contracts with Acumen, ADx Neurosciences, AC‐Immune, Alamar, Aribio, Axon Neurosciences, Beckman‐Coulter, BioConnect, Bioorchestra, Brainstorm Therapeutics, Celgene, Cognition Therapeutics, EIP Pharma, Eisai, Eli Lilly, Fujirebio, Grifols, Instant Nano Biosensors, Merck, Novo Nordisk, Olink, PeopleBio, Quanterix, Roche, Siemens, Toyama, and Vivoryon. Charlotte E. Teunissen is editor‐in‐chief of *Alzheimer Research and Therapy* and serves on editorial boards of Medidact Neurologie/Springer, and *Neurology: Neuroimmunology & Neuroinflammation*. Charlotte E. Teunissen had speaker contracts for Eli Lilly, Novo Nordisk, Olink, and Roche. Agnes Flöel had speaker contracts for Eli Lilly, Biogen Idec, Eisai, and Roche, and advisory board contracts for Eli Lilly and Biogen Idec. Sylvain Lehmann has speaker contracts with Eli Lilly, Roche, Beckman Coulter, and Fujirebio, and advisory board contracts with Beckman Coulter and Roche. Péter Körtvélyessy consulted Biogen, Eli Lilly, and Novartis; had speaker contracts with Eisai, Eli Lilly, and Novartis;, and advisory board contracts with Eli Lilly and Biogen. There is nothing to disclose for Andrea Dell'Orco, Ariane Fillmer, Claudia Schwarz, Jeanette Melin, Laura Göschel, Layla Riemann, Leslie Pendrill, Patty L. Hoede, Semiha Aydin, Stefan Cano, and Ulrike Grittner. Author disclosures are available in the supporting information.

## CONSENT STATEMENT

All participants gave written informed consent before participating in the study.

## Supporting information



Supporting Information

Supporting Information
