# Supplementary material for: Plasma p‐tau181 and GFAP reflect 7T MR‐derived changes in Alzheimer's disease: A longitudinal study of structural and functional MRI and MRS
Source: Alzheimers Dement. 2024 Nov 19;20(12):8684–99. doi: 10.1002/alz.14318 (PMC11667506; doi:10.1002/alz.14318)
Supplement: Supplementary file 1 — Supporting Information [file ALZ-20-8684-s001.docx]

#### Appendix A

**Model descriptions:**

Model 1:

plasma_biomarker_ij_ or outcome_ij_= β_0_ + u_0i_ + β_1_*diagnosis_i_*time_ij_ + β_2_*age_ij_ + β_3_*diagnosis_i_ + β_4_*time_ij_ + ε_ij_

Model 2:

Z_outcome_ij_ = β_0_ + u_0i_ + β_1_*Z_plasma_biomarker_ij_ *time_ij_ + β_2_*age_ij_ + β_3_*sex_i_ + β_4_*education_i_ + β_5_*Z_plasma_biomarker_ij_ + β_6_*time_ij_ + ε_ij_

with β_0_: intercept, u_0i_: residual for intercept for participant i, Z_plasma_biomarker_ij_: (Z-scaled) plasma biomarker concentration of participant i at time j, diagnosis_j_: diagnosis of participant i at visit 1, time_ij_: time point of measure at time j for participant i, age_ij_: age of participant i at time j, ε_ij_: residual for participant i at time j, Z_outcome_ij_: z-scaled MR-based measurements or cognition for participant i at time j, sex_i_: sex of participant I, education_i_: years of education of participant i.

**Exploration of the study sample:**

The study sample was grouped into diagnoses (HC, SCD, MCI, AD, figure A1).


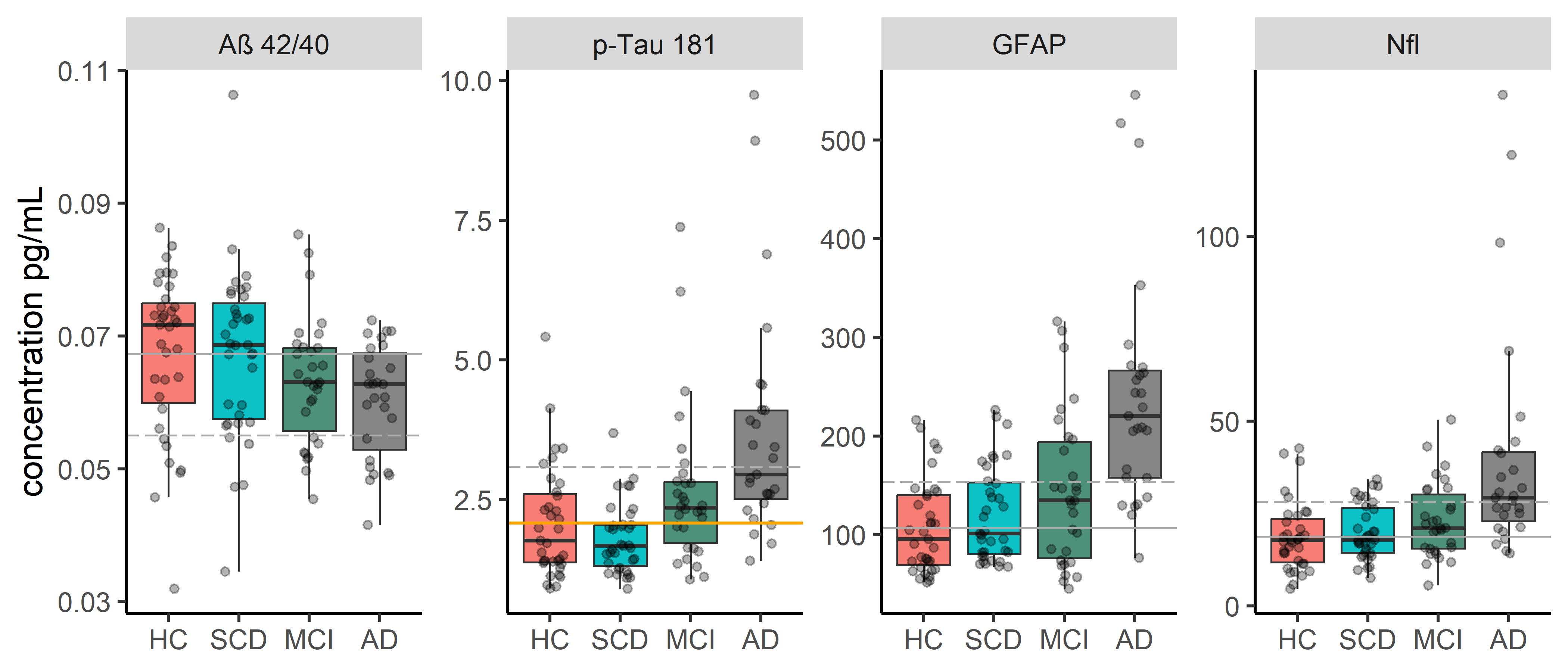


**Figure A1: Concentration of plasma biomarkers at visit 1 by diagnosis.** Gray horizontal lines indicate the mean of the HC group (continuous line) and 1sd towards the pathological direction (long dashed line). The orange horizontal line represents a threshold for elevated plasma p-Tau 181 (>2.08 pg/mL), which coincides with the mean for plasma p-Tau 181. Further analyses using the plasma p-Tau status are shown in the end of the appendix. Abbreviations: AD, Alzheimer’s disease; Aß, amyloid beta; GFAP, glial fibrillary acidic protein; HC, healthy control; MCI, mild cognitive impairment; NfL, neurofilament light chain; p-Tau 181, tau phosphorylated at threonine 181; SCD, subjective cognitive decline.


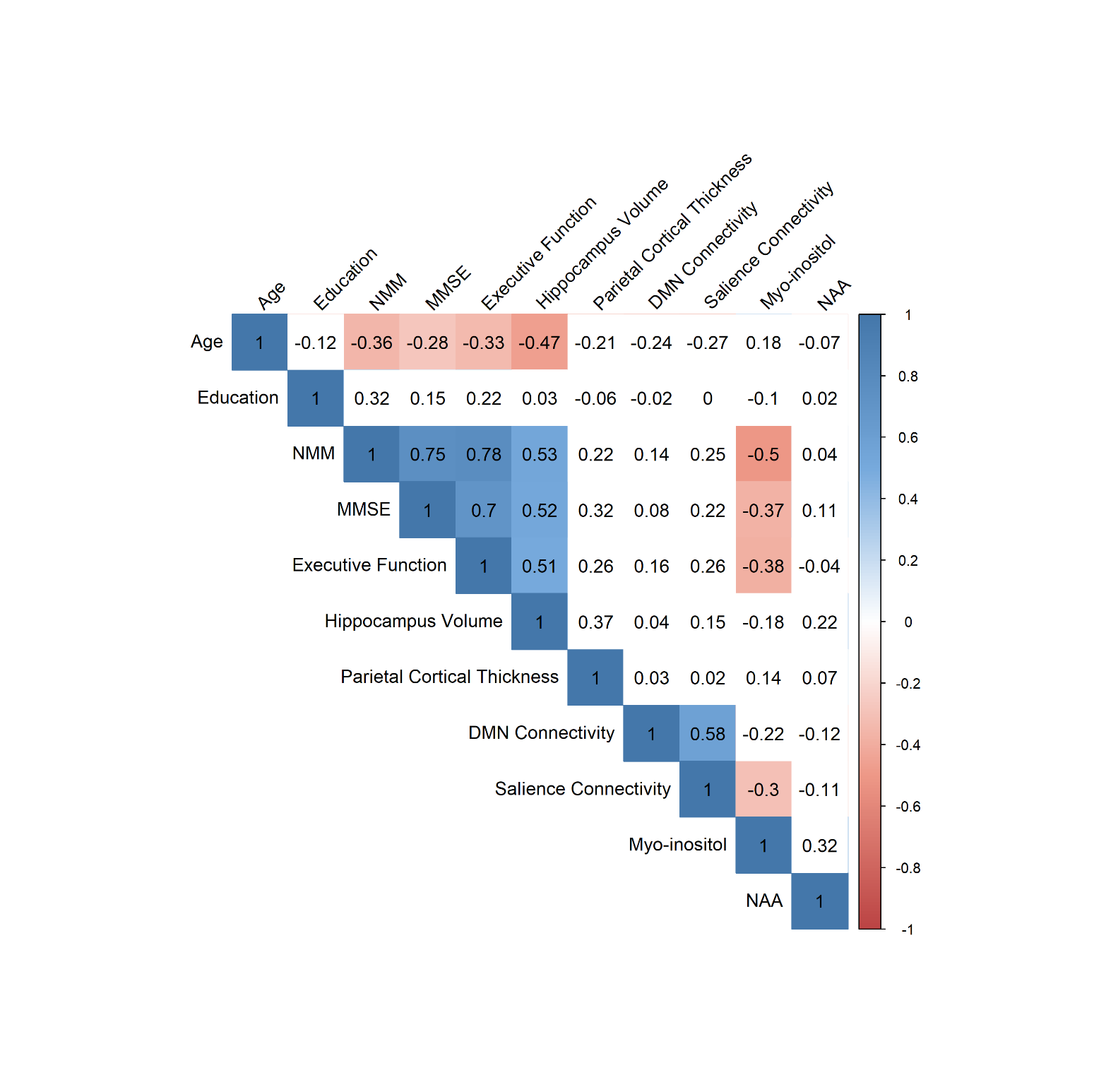


**Figure A2: Correlation matrix of imaging biomarkers at visit 1.** Correlation coefficients were displayed numerically and using a color scale. Non-significant correlations (p > 0.05) were not colored. Note that higher myo-inositol indicates neuroinflammation and lower NAA indicates less neuronal integrity. Abbreviations: NAA, N-Acetylaspartic acid; NMM, NeuroMET Memory Metric; MMSE, Mini-Mental State Examination; DMN, Default Mode Network; Sal, Salience Connectivity.

**Table A1: Associations between concentrations of plasma Aß42/40, p-Tau 181, GFAP, and NfL and measurements of cognition, structural and functional MRI and MRS at visit 1.**

|  |  | **Aß 42/40** | |  | **p-Tau 181** | |  | **GFAP** | |  | **NfL** | |
| --- | --- | --- | --- | --- | --- | --- | --- | --- | --- | --- | --- | --- |
| z-scores | n | std. ß [95% CI] | p |  | std. ß [95% CI] | p |  | std. ß [95% CI] | p |  | std. ß [95% CI] | p |
| NMM | 127 | 0.20 [-0.14; 0.55] | .242 |  | -0.44 [-0.66; -0.22] | **<.001** |  | -0.65 [-0.81; -0.50] | **<.001** |  | -0.49 [-0.65; -0.32] | **<.001** |
| Executive Function | 126 | 0.18 [0.01; 0.35] | **.033** |  | -0.18 [-0.29; -0.06] | **.003** |  | -0.28 [-0.37; -0.19] | **<.001** |  | -0.21 [-0.30; -0.12] | **<.001** |
| Hippocampus Volume | 111 | 0.06 [-0.08; 0.20] | .412 |  | -0.06 [-0.16; 0.04] | .214 |  | -0.15 [-0.24; -0.07] | **.001** |  | -0.04 [-0.13; 0.05] | .377 |
| Parietal Cortical Thickness | 111 | 0.08 [-0.16; 0.32] | .523 |  | -0.14 [-0.3; 0.03] | .097 |  | -0.16 [-0.28; -0.04] | **.011** |  | -0.11 [-0.22; 0.01] | .071 |
| DMN Connectivity | 114 | -0.14 [-0.32; 0.05] | .146 |  | -0.11 [-0.24; 0.02] | .100 |  | -0.06 [-0.16; 0.04] | .238 |  | -0.03 [-0.14; 0.08] | .581 |
| Sal Connectivity | 114 | -0.14 [-0.32; 0.04] | .132 |  | -0.21 [-0.34; -0.09] | **.001** |  | -0.07 [-0.17; 0.03] | .168 |  | -0.06 [-0.17; 0.05] | .276 |
| Myo-inositol | 117 | -0.06 [-0.32; 0.19] | .639 |  | 0.24 [0.08; 0.41] | **.005** |  | 0.23 [0.10; 0.36] | **.001** |  | 0.20 [0.07; 0.32] | **.003** |
| NAA | 117 | 0.18 [0.00; 0.37] | .056 |  | -0.06 [-0.19; 0.07] | .338 |  | -0.16 [-0.26; -0.06] | **.002** |  | -0.11 [-0.21; -0.01] | **.028** |
| The effects [95% CI] were estimated by linear mixed models adjusted for age, sex, and education. Models including the MRS markers myo-inositol and NAA were additionally weighted based on a measure of uncertainty (CRLB). All plasma biomarkers and outcome variables were z-standardized to allow for comparisons. *Abbreviations: Aß, amyloid beta; CI, confidence interval; CRLB, Cramer-Rao Lower Bound; DMN, default mode network; GFAP, glial fibrillary acidic protein; NAA, N-Acetylaspartic acid; NfL, neurofilament light chain; NMM, NeuroMET Memory Metric; p-Tau 181, phosphorylated tau at threonine 181; Sal, Salience network.* | | | | | | | | | | | | |

**Table A2: Interaction effects of plasma Aß42/40, p-Tau 181, GFAP and NfL over time on changes of measurements of cognition, structural and functional MRI and MRS**. Positive effects can be interpreted as stronger changes in the AD-related outcome over time, while negative effects explain a weakening of the effects over time. The interaction effects do not contain any information on the directionality of the effects of plasma biomarker concentration on MR-based measurements and have to be interpreted together with the main effect, which is why we provide effects for discrete changes of plasma biomarker concentration in the main text and table A4.

|  |  |  | Aß 42/40 x time | |  | p-Tau 181 x time | |  | GFAP x time | |  | NfL x time | |
| --- | --- | --- | --- | --- | --- | --- | --- | --- | --- | --- | --- | --- | --- |
| z-scores | n obs. | n  part. | std. ß [95% CI] | p |  | std. ß [95% CI] | p |  | std. ß [95% CI] | p |  | std. ß [95% CI] | p |
| NMM | 243 | 127 | 0.07 [-0.04; 0.17] | .236 |  | -0.15 [-0.24; -0.06] | **.001** |  | -0.06 [-0.12; 0.00] | .057 |  | 0.01 [-0.06; 0.08] | .736 |
| Executive Function | 242 | 126 | 0.02 [-0.02; 0.06] | .387 |  | 0.00 [-0.04; 0.03] | .836 |  | 0.00 [-0.03; 0.03] | .995 |  | 0.01 [-0.02; 0.03] | .625 |
| Hippocampus Volume | 197 | 111 | 0.03 [0.01; 0.06] | **.018** |  | -0.02 [-0.04; 0.01] | .241 |  | -0.03 [-0.04; -0.01] | **.004** |  | -0.01 [-0.03; 0.01] | .449 |
| Parietal Cortical Thickness | 196 | 111 | 0.03 [-0.06; 0.11] | .482 |  | -0.04 [-0.11; 0.03] | .272 |  | 0.01 [-0.05; 0.06] | .825 |  | 0.02 [-0.03; 0.08] | .402 |
| DMN Connectivity | 201 | 114 | 0.04 [-0.04; 0.13] | .313 |  | 0.03 [-0.05; 0.10] | .480 |  | 0.01 [-0.04; 0.06] | .726 |  | 0.00 [-0.05; 0.06] | .973 |
| Sal Connectivity | 201 | 114 | 0.11 [0.03; 0.19] | **.008** |  | -0.04 [-0.11; 0.03] | .308 |  | -0.04 [-0.09; 0.02] | .145 |  | -0.03 [-0.08; 0.02] | .243 |
| Myo-inositol | 214 | 117 | -0.03 [-0.08; 0.02] | .512 |  | 0.01 [-0.04; 0.07] | .802 |  | 0.01 [-0.03; 0.05] | .691 |  | -0.05 [-0.08; -0.01] | .147 |
| NAA | 214 | 117 | -0.03 [-0.03; 0.00] | .493 |  | 0.05 [0.03; 0.07] | .168 |  | 0.05 [0.05; 0.08] | **.040** |  | 0.02 [0.02; 0.05] | .314 |
| The effects [95% CI] were estimated by linear mixed models adjusted for age, sex, and education. Models including the MRS markers myo-inositol and NAA were additionally weighted based on a measure of uncertainty (CRLB). All plasma biomarkers, MR-based, and cognition measurements were z-standardized to allow for comparisons. *Abbreviations: Aß, amyloid beta; CI, confidence interval; CRLB, Cramer-Rao Lower Bound; DMN, Default Mode Network; GFAP, glial fibrillary acidic protein; NAA, N-Acetylaspartic acid; NfL, neurofilament light chain; NMM, NeuroMET Memory Metric; obs., observations; part., participants; p-Tau 181, tau phosphorylated at threonine 181; Sal, Salience network.* | | | | | | | | | | | | | |

**Table A3: Yearly changes (z-score) of measurements of cognition, structural and functional MRI, and MRS estimated for discrete changes of plasma biomarker concentration for Aß42/40, p-Tau 181, GFAP and NfL (normal = HC mean, abnormal = HC mean +/- 1sd towards the pathological direction).**

|  |  |  | normal stable | |  | abnormal stable | |  | normal => abnormal | |
| --- | --- | --- | --- | --- | --- | --- | --- | --- | --- | --- |
|  | n obs. | n part. | yearly change  (z-score) | p |  | yearly change  (z-score) | p |  | yearly change  (z-score) | p |
| **Aß42/40** |  |  |  |  |  |  |  |  |  |  |
| NMM | 243 | 127 | 0.10 [-0.04; 0.23] | .240 |  | 0.03 [-0.15; 0.22] | .968 |  | -0.17 [-0.62; 0.28] | .759 |
| Executive Function | 242 | 126 | 0.00 [-0.06; 0.06] | 1.000 |  | -0.02 [-0.09; 0.05] | .901 |  | -0.20 [-0.42; 0.02] | .085 |
| Hippocampus Volume | 197 | 111 | 0.03 [-0.03; 0.09] | .519 |  | 0.00 [-0.07; 0.06] | 1.000 |  | -0.06 [-0.26; 0.13] | .841 |
| Parietal Cortical Thickness | 196 | 111 | -0.10 [-0.20; 0.01] | **.081** |  | -0.13 [-0.27; 0.02] | .105 |  | -0.21 [-0.52; 0.11] | .337 |
| DMN Connectivity | 201 | 114 | -0.04 [-0.14; 0.05] | .658 |  | -0.09 [-0.23; 0.06] | .406 |  | 0.05 [-0.19; 0.30] | .948 |
| Sal Connectivity | 201 | 114 | -0.09 [-0.19; 0.00] | .066 |  | -0.20 [-0.34; -0.06] | **.002** |  | -0.06 [-0.30; 0.18] | .902 |
| Myo-inositol | 170 | 127 | -0.02 [-0.14; 0.10] | .956 |  | 0.01 [-0.16; 0.17] | .999 |  | 0.07 [-0.27; 0.41] | .952 |
| NAA | 214 | 117 | 0.02 [-0.07; 0.11] | .900 |  | 0.05 [-0.08; 0.18] | .736 |  | -0.13 [-0.38; 0.11] | .502 |
| **p-Tau 181** |  |  |  |  |  |  |  |  |  |  |
| NMM | 243 | 127 | 0.13 [0.00; 0.27] | **.043** |  | -0.02 [-0.17; 0.13] | .991 |  | -0.45 [-0.75; -0.16] | **<.001** |
| Executive Function | 242 | 126 | 0.00 [-0.06; 0.05] | .998 |  | -0.01 [-0.07; 0.06] | .989 |  | -0.18 [-0.34; -0.03] | **.011** |
| Hippocampus Volume | 197 | 111 | 0.03 [-0.03; 0.09] | .545 |  | 0.01 [-0.05; 0.08] | .933 |  | -0.05 [-0.18; 0.09] | .786 |
| Parietal Cortical Thickness | 196 | 111 | -0.09 [-0.20; 0.02] | **.148** |  | -0.13 [-0.25; -0.01] | **.028** |  | -0.27 [-0.50; -0.04] | **.014** |
| DMN Connectivity | 201 | 114 | -0.06 [-0.16; 0.04] | .451 |  | -0.03 [-0.15; 0.09] | .902 |  | -0.14 [-0.34; 0.05] | .232 |
| Sal Connectivity | 201 | 114 | -0.09 [-0.19; 0.00] | .066 |  | -0.13 [-0.25; -0.02] | **.020** |  | -0.34 [-0.53; -0.16] | **<.001** |
| Myo-inositol | 170 | 127 | -0.02 [-0.14; 0.10] | .977 |  | -0.01 [-0.15; 0.14] | .999 |  | 0.24 [-0.01; 0.48] | .063 |
| NAA | 214 | 117 | 0.01 [-0.08; 0.11] | .988 |  | 0.06 [-0.05; 0.17] | .499 |  | -0.01 [-0.19; 0.19] | 1.000 |
| **GFAP** |  |  |  |  |  |  |  |  |  |  |
| NMM | 243 | 127 | 0.13 [0.00; 0.26] | .059 |  | 0.07 [-0.05; 0.19] | .483 |  | -0.59 [-0.82; -0.35] | **<.001** |
| Executive Function | 242 | 126 | 0.00 [-0.06; 0.05] | .998 |  | 0.00 [-0.06; 0.05] | .997 |  | -0.29 [-0.42; -0.16] | **<.001** |
| Hippocampus Volume | 197 | 111 | 0.04 [-0.01; 0.10] | .220 |  | 0.02 [-0.04; 0.07] | .892 |  | -0.14 [-0.26; -0.01] | **.023** |
| Parietal Cortical Thickness | 196 | 111 | -0.10 [-0.22; 0.01] | **.097** |  | -0.10 [-0.21; 0.01] | .094 |  | -0.25 [-0.44; -0.07] | **.003** |
| DMN Connectivity | 201 | 114 | -0.05 [-0.16; 0.05] | .532 |  | -0.05 [-0.15; 0.06] | .653 |  | -0.11 [-0.27; 0.06] | .334 |
| Sal Connectivity | 201 | 114 | -0.08 [-0.18; 0.02] | .196 |  | -0.12 [-0.22; -0.02] | **.012** |  | -0.19 [-0.35; -0.03] | **.014** |
| Myo-inositol | 170 | 127 | -0.02 [-0.15; 0.11] | .966 |  | -0.01 [-0.13; 0.11] | .996 |  | 0.22 [-0.01; 0.42] | **.036** |
| NAA | 214 | 117 | -0.01 [-0.11; 0.09] | .991 |  | 0.04 [-0.05; 0.13] | .685 |  | -0.12 [-0.28; 0.03] | .182 |
| **NfL** |  |  |  |  |  |  |  |  |  |  |
| NMM | 243 | 127 | 0.09 [-0.04; 0.23] | .294 |  | 0.10 [-0.03; 0.24] | .193 |  | -0.38 [-0.62; -0.15] | **<.001** |
| Executive Function | 242 | 126 | 0.00 [-0.06; 0.05] | .999 |  | 0.00 [-0.05; 0.06] | .999 |  | -0.21 [-0.33; -0.09] | **<.001** |
| Hippocampus Volume | 197 | 111 | 0.03 [-0.03; 0.09] | .499 |  | 0.02 [-0.04; 0.08] | .757 |  | -0.02 [-0.13; 0.10] | .984 |
| Parietal Cortical Thickness | 196 | 111 | -0.11 [-0.22; -0.00] | **.053** |  | -0.09 [-0.20; 0.03] | .208 |  | -0.19 [-0.37; -0.02] | **.027** |
| DMN Connectivity | 201 | 114 | -0.05 [-0.15; 0.05] | .600 |  | -0.05 [-0.15; 0.06] | .640 |  | -0.08 [-0.25; 0.09] | .621 |
| Sal Connectivity | 201 | 114 | -0.09 [-0.19; 0.01] | .110 |  | -0.12 [-0.22; -0.01] | **.018** |  | -0.18 [-0.35; -0.01] | **.030** |
| Myo-inositol | 170 | 127 | 0.00 [-0.12; 0.13] | 1.000 |  | -0.05 [-0.17; 0.08] | .794 |  | 0.15 [-0.05; 0.35] | .200 |
| NAA | 214 | 117 | 0.01 [-0.08; 0.11] | .979 |  | 0.04 [-0.06; 0.13] | .716 |  | -0.07 [-0.22; 0.08] | .613 |
| Yearly changes were estimated by linear mixed models adjusted for age, sex, and education. Models including the MRS markers myo-inositol and NAA were additionally weighted based on a measure of uncertainty (CRLB). All plasma biomarkers, MR-based, and cognition measurements were z-standardized to allow for comparisons. *Abbreviations: Aß, amyloid beta; CI, confidence interval; CRLB, Cramer-Rao Lower Bound; DMN, Default Mode Network; GFAP, glial fibrillary acidic protein; HC, healthy control; NAA, N-Acetylaspartic acid; NfL, neurofilament light chain; NMM, NeuroMET Memory Metric; obs., observations; part., participants; p-Tau 181, tau phosphorylated at threonine 181; Sal, Salience network.* | | | | | | | | | | |

**Exploratory Analysis classifying the Study Sample using a Threshold for Plasma p-Tau 181:**

In response to emerging suggestions for utilizing plasma p-Tau levels as a biomarker for Alzheimer's disease staging [1], we calculated a threshold to classify individual p-Tau181 status into positive (p-Tau+ >2.08 pg/mL) and negative status. The p-Tau 181 threshold indicates amyloid pathology and was established in a previous study by the neurochemistry laboratory, Amsterdam UMC, Amsterdam [2]. The threshold value was adapted to the kit lot used in the current study, using 10 bridging samples that were re-measured. Figure A3 presents the cross-sectional and longitudinal concentrations of the plasma biomarkers Aß42/40, GFAP and NfL grouped by p-Tau181 status at visit 1.

***
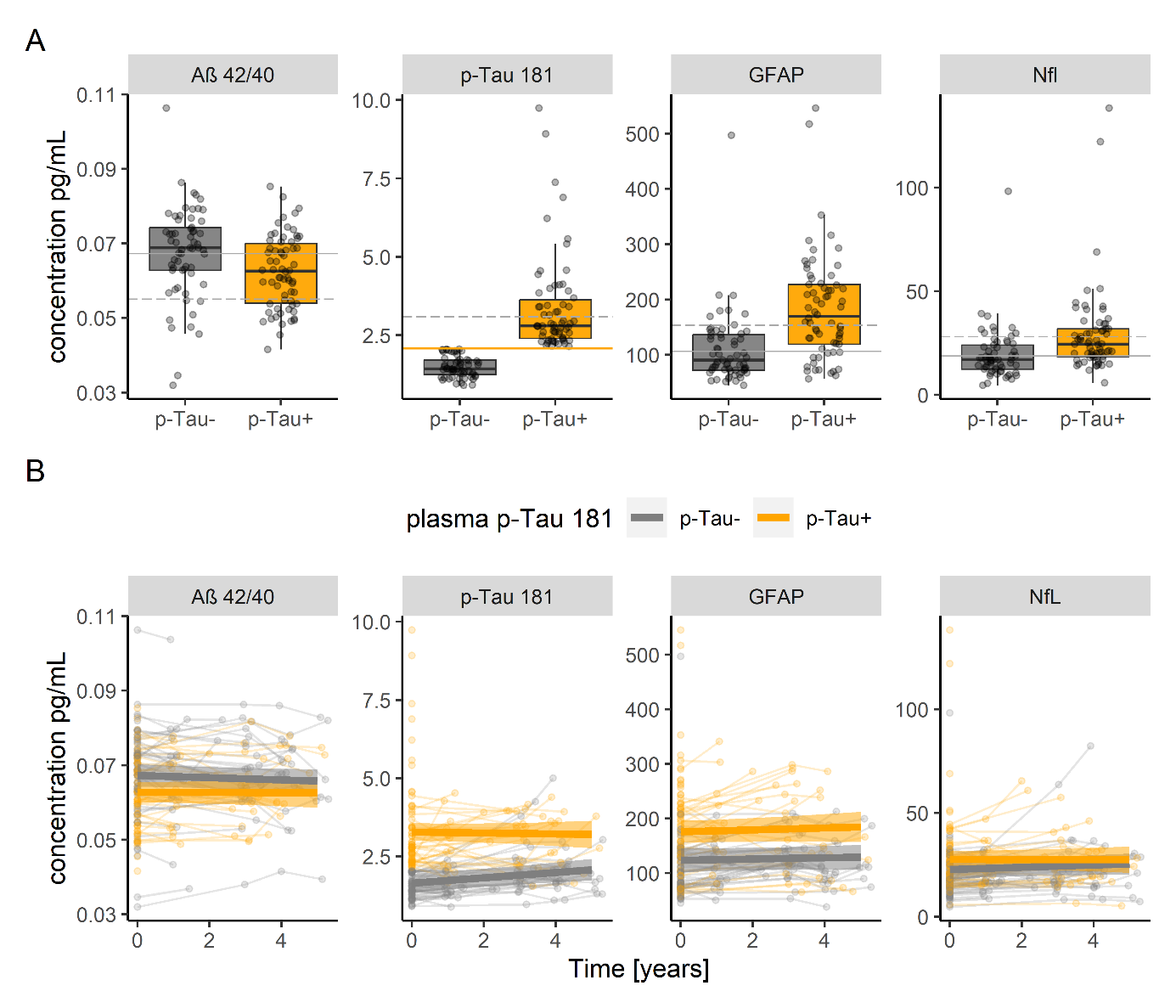
***

**Figure A3: Concentrations of plasma biomarkers by plasma p-Tau 181 status (abnormal/positive** **>2.08 pg/mL) at visit 1 (A) and longitudinal (B).** *Abbreviations: Aß, amyloid beta; GFAP, glial fibrillary acidic protein; NfL, neurofilament light chain; p-Tau 181, tau phosphorylated at threonine 181.*

This threshold was applied to re-run our cross-sectional analyses to assess how plasma p-Tau181 status influences the association between plasma Aß42/40, GFAP and NfL and 7T MRI/MRS-derived parameters.

Model:

Z_outcome _ij_ = β_0_ + u_0i_ + β_1_*Z_plasma_biomarker_ij_ *time_ij_*p-Tau_status_ij_ + β_2_*age_ij_ + β_3_*sex_i_ + β_4_*education_i_ + β_5_*Z_plasma_biomarker_ij_ + β_6_*time_ij_ + β_7_* p-Tau_status_ij_ + ε_ij_

The cross-sectional analyses showed that higher levels of GFAP were significantly associated with smaller hippocampus volume only among individuals classified as p-Tau+ (std. ß [95% CI] = -0.18 [-0.29; -0.07]) but not p-Tau- (std. ß [95% CI] = 0.01 [-0.15; 0.17], ß_difference_ = -0.19, p_difference_ = 0.044, figure A4, table A4), which confirms GFAP’s role in AD pathology versus normal aging. Further, p-Tau status had no significant effect on the other associations, likely due to limited sample size, as indicated by the large confidence intervals. Analysis on the longitudinal associations was not pursued as the sample size did not provide sufficient power for reliable conclusions.


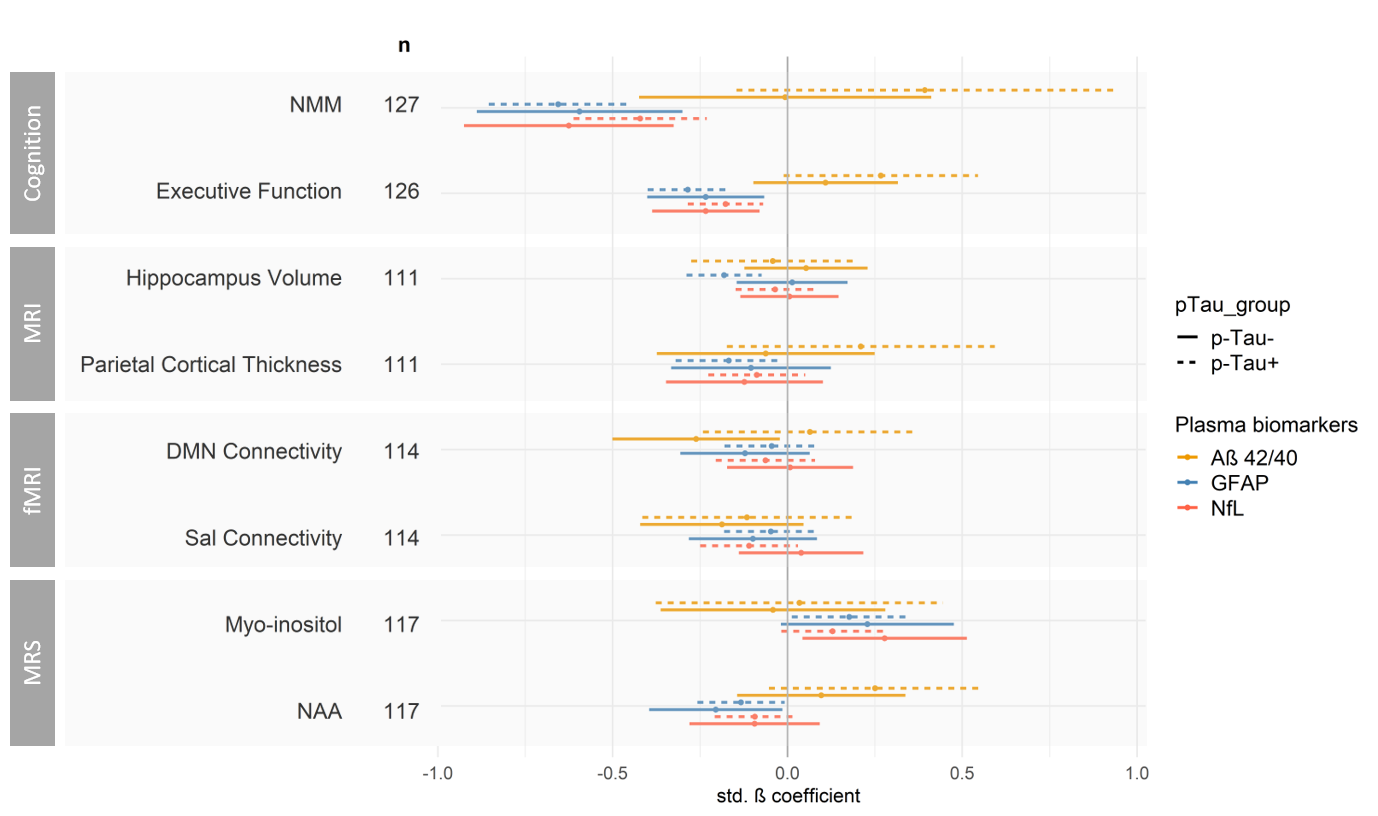


**Figure A4: Cross-sectional associations between concentrations of plasma Aß42/40, GFAP and NfL and z-transformed parameters of cognition, structural and functional MRI, and MRS (at visit 1) grouped by plasma p-Tau181 status (p-Tau+ >2.08 pg/mL).** Increased GFAP was associated with smaller hippocampus volume only for individuals with p-Tau+ (std. ß [95% CI] = -0.18 [-0.29; -0.07]) but not p-Tau- (std. ß [95% CI] = 0.01 [-0.15; 0.17], ß difference = -0.19, p = 0.044). P-Tau status did not show any significant interaction for the other associations most likely due to limited power indicated by large confidence intervals. Depending on data availability, observations of a maximum of 127 participants were included. The effects are presented with 95% CI (horizontal bars) estimated by linear mixed models adjusted for age, sex, and education. Models including the MRS parameters myo-inositol and NAA were additionally weighted based on a measure of uncertainty (CRLB). Connectivity measures for the DMN and Sal were acquired at resting-state. *Abbreviations: Aß, amyloid ß; CI, confidence interval; CRLB, Cramer-Rao Lower Bound; DMN, Default mode network; fMRI, functional magnetic resonance imaging; GFAP, glial fibrillary acidic protein; MRI, magnetic resonance imaging; MRS, magnetic resonance spectroscopy; NAA, N-Acetylaspartic acid; NfL, neurofilament light chain; NMM, NeuroMET memory metric; p-Tau 181, tau phosphorylated at threonine 181; Sal, Salience network.*

**Table A4: Interaction effects of plasma p-Tau 181 status** **(p-Tau+ >2.08 pg/mL) on the association between plasma Aß42/40, GFAP and NfL on measurements of cognition, structural and functional MRI and MRS**.

|  | n | p-Tau- std. ß [95% CI] |  | p-Tau+ std. ß [95% CI] | Diff | p |  |
| --- | --- | --- | --- | --- | --- | --- | --- |
| **Aß42/40** |  |  |  |  |  |  |  |
| NMM | 127 | -0.01 [-0.42; 0.41] |  | 0.39 [-0.15; 0.93] | 0.40 | 0.247 |  |
| Executive Function | 126 | 0.11 [-0.10; 0.32] |  | 0.27 [-0.01; 0.55] | 0.16 | 0.368 |  |
| Hippocampus Volume | 111 | 0.05 [-0.12; 0.23] |  | -0.04 [-0.28; 0.19] | -0.10 | 0.519 |  |
| Parietal Cortical Thickness | 111 | -0.06 [-0.37; 0.25] |  | 0.21 [-0.17; 0.59] | 0.27 | 0.277 |  |
| DMN Connectivity | 114 | -0.26 [-0.50; -0.02] |  | 0.06 [-0.24; 0.37] | 0.33 | 0.098 |  |
| Sal Connectivity | 114 | -0.19 [-0.42; 0.05] |  | -0.12 [-0.42; 0.18] | 0.07 | 0.709 |  |
| Myo-inositol | 117 | -0.04 [-0.36; 0.28] |  | 0.03 [-0.38; 0.44] | 0.08 | 0.774 |  |
| NAA | 117 | 0.10 [-0.14; 0.34] |  | 0.25 [-0.05; 0.55] | 0.15 | 0.432 |  |
| **GFAP** |  |  |  |  |  |  |  |
| NMM | 127 | -0.59 [-0.89; -0.30] |  | -0.66 [-0.85; -0.46] | -0.06 | 0.725 |  |
| Executive Function | 126 | -0.23 [-0.40; -0.07] |  | -0.28 [-0.40; -0.17] | -0.05 | 0.611 |  |
| Hippocampus Volume | 111 | 0.01 [-0.15; 0.17] |  | -0.18 [-0.29; -0.07] | -0.19 | **0.044** |  |
| Parietal Cortical Thickness | 111 | -0.10 [-0.33; 0.12] |  | -0.17 [-0.32; -0.02] | -0.06 | 0.640 |  |
| DMN Connectivity | 114 | -0.12 [-0.31; 0.06] |  | -0.05 [-0.18; 0.09] | 0.08 | 0.502 |  |
| Sal Connectivity | 114 | -0.10 [-0.28; 0.08] |  | -0.05 [-0.18; 0.09] | 0.05 | 0.649 |  |
| Myo-inositol | 117 | 0.23 [-0.02; 0.48] |  | 0.18 [0.01; 0.34] | -0.05 | 0.724 |  |
| NAA | 117 | -0.21 [-0.40; -0.01] |  | -0.13 [-0.26; -0.01] | 0.07 | 0.521 |  |
| **NfL** |  |  |  |  |  |  |  |
| NMM | 127 | -0.63 [-0.93; -0.33] |  | -0.42 [-0.61; -0.23] | 0.20 | 0.248 |  |
| Executive Function | 126 | -0.23 [-0.39; -0.08] |  | -0.18 [-0.29; -0.07] | 0.06 | 0.546 |  |
| Hippocampus Volume | 111 | 0.01 [-0.13; 0.15] |  | -0.04 [-0.15; 0.08] | -0.04 | 0.652 |  |
| Parietal Cortical Thickness | 111 | -0.12 [-0.35; 0.10] |  | -0.09 [-0.23; 0.05] | 0.04 | 0.787 |  |
| DMN Connectivity | 114 | 0.01 [-0.17; 0.19] |  | -0.06 [-0.20; 0.08] | -0.07 | 0.533 |  |
| Sal Connectivity | 114 | 0.04 [-0.14; 0.22] |  | -0.11 [-0.25; 0.03] | -0.15 | 0.183 |  |
| Myo-inositol | 117 | 0.28 [0.04; 0.51] |  | 0.13 [-0.02; 0.28] | -0.15 | 0.278 |  |
| NAA | 117 | -0.09 [-0.28; 0.09] |  | -0.09 [-0.21; 0.02] | 0.00 | 0.996 |  |
| The effects [95% CI] were estimated by linear mixed models adjusted for age, sex, and education. Models including the MRS markers myo-inositol and NAA were additionally weighted based on a measure of uncertainty (CRLB). All plasma biomarkers and outcome variables were z-standardized to allow for comparisons. *Abbreviations: Aß, amyloid beta; CI, confidence interval; CRLB, Cramer-Rao Lower Bound; DMN, default mode network; GFAP, glial fibrillary acidic protein; NAA, N-Acetylaspartic acid; NfL, neurofilament light chain; NMM, NeuroMET Memory Metric; p-Tau 181, phosphorylated tau at threonine 181; Sal, Salience network.* | | | | | | |  |

[1] Jack CR, Jr., Andrews JS, Beach TG, Buracchio T, Dunn B, Graf A, et al. Revised criteria for diagnosis and staging of Alzheimer's disease: Alzheimer's Association Workgroup. Alzheimers Dement. 2024; <https://doi.org/10.1002/alz.13859>

[2] Verberk IMW, Jutte J, Kingma MY, Vigneswaran S, Gouda M, van Engelen MP, et al. Development of thresholds and a visualization tool for use of a blood test in routine clinical dementia practice. Alzheimers Dement. 2024; <https://doi.org/10.1002/alz.14088>
